# Evaluating the effects of ultrasonic and high-pressure homogenization on the flavor profile of Wuliang Mountain black-boned chicken soup

**DOI:** 10.1016/j.ultsonch.2026.107810

**Published:** 2026-03-04

**Authors:** Shuaihan Jiang, Hongqin Chen, Sisi Zhang, Zhiqiang Xu, Shijun Li

**Affiliations:** College of Food Science and Technology, Yunnan Agricultural University, Kunming 650201, China

**Keywords:** Wuliang Mountain black-boned chicken, Ultrasound, High-pressure homogenization, Food processing

## Abstract

This study evaluated conventional stewing (C), ultrasonic (U), and high-pressure homogenization (H) processing of Wuliang Mountain black-boned chicken soup. The U group achieved superior sensory scores and higher levels of key flavor compounds like aldehydes, esters, and ketones, also showing the highest odor activity value. Ultrasonic treatment significantly increased umami and sweet amino acids. The H group exhibited elevated free fatty acids, acids, and phenols. Among 107 volatile compounds identified, 57 had high discriminatory power (VIP > 1), with five markers unique to the U and H groups. Correlations showed amino acids and fatty acids significantly influence flavor. Ultrasonic treatment best enhanced overall sensory quality and flavor, while high-pressure homogenization effectively increased free fatty acids, supporting better utilization of this chicken breed.

## Highlights

• This study represents the first application of combined ultrasound-assisted stewing and high-pressure homogenization to process Wuliang Mountain black-boned chicken broth.
• The sensory quality of the chicken soup was significantly improved and the content of flavor compounds was increased by ultrasonic treatment.
• The free fatty acids (FFAs) content was significantly increased by high-pressure homogenization treatment.
• A scientific basis for the resource development, utilization, and preservation of Wuliang Mountain black-boned chicken was provided by this research.

## Graphical abstract

**Figure:**
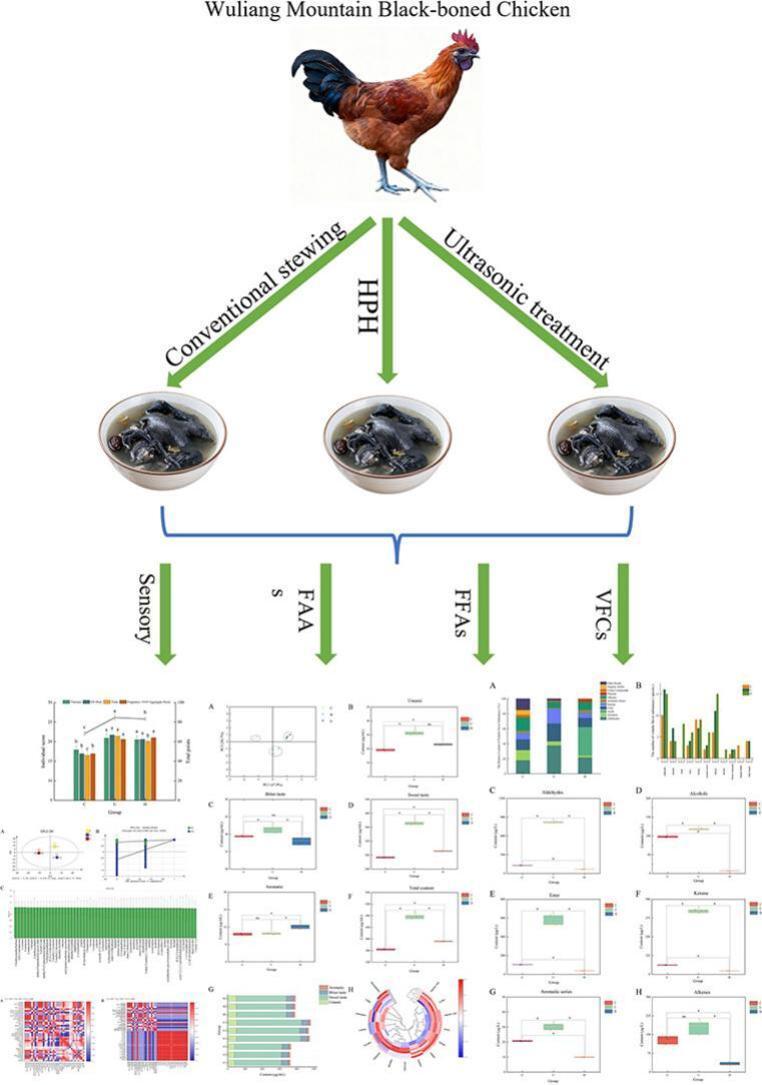

## 1 Introduction

Chicken soup is a source of essential nutrients, including proteins, amino acids, vitamins, and trace elements [1], and is widely valued as a nutritious tonic. It has also been associated with health benefits such as blood sugar reduction and immune function enhancement [2]. During the cooking process, flavor precursors from the chicken meat—such as proteins, free amino acids (FAAs), reducing sugars, nucleotides, and unsaturated fatty acids—dissolve into the broth. The presence of these compounds contributes significantly to the soup's desirable flavor and nutritional profile [3]. The Wuliang Mountain black-boned chicken, an indigenous breed from Yunnan Province in southwestern China, is renowned for its favorable meat traits and substantial economic value [4]. This breed was officially listed in the National Catalog of Livestock and Poultry Genetic Resources by the Ministry of Agriculture in 2010 and was awarded protected geographical indication status in 2013. This breed was developed through the long-term domestication and selection of red junglefowl by indigenous communities in the Wuliang Mountain range, resulting in distinct physiological and nutritional traits. It is characterized by a large body size, robustness, strong adaptability, firm yet tender meat, and the distinctive black pigmentation of its bones, flesh, and internal organs. Currently, scientific research on the Wuliang Mountain black-boned chicken is predominantly conducted within the field of animal science. For instance, polymorphisms in the PITX2 exon were identified by Cao et al. [5] as significantly influencing the body and carcass traits of this breed. In a subsequent study, Cao et al. [6] investigated the expression of PITX2 and SIX1 in the pectoral muscle, revealing that their expression levels were significantly correlated with key meat quality attributes, including color, tenderness, pH, and drip loss.

The development of flavor in traditional chicken soup involves a complex series of chemical reactions, primarily including protein degradation, lipid oxidation, and the Maillard reaction. The characteristic umami taste is largely derived from FAAs such as glutamic acid and aspartic acid [7], as well as flavor nucleotides like inosine monophosphate. During boiling, the oxidation of chicken fat generates lipid-derived flavor compounds, while amino acids and sugars undergo the Maillard reaction at high temperatures to form aromatic substances [8]. As an emerging technology, ultrasound can significantly accelerate food processing while preserving product quality. This effect is attributed to cavitation, which generates hydrogen peroxide that induces the oxidation of meat proteins and lipids [9]. It was found that pretreatment with ultrasound influences small molecule metabolites and flavor compounds in chicken soup, leading to an improvement in its overall taste and flavor profile [10]. Furthermore, ultrasound-assisted stewing was reported to enhance the nutritional concentration, emulsifying properties, and flavor characteristics of chicken soup. The relative concentration of marker volatile organic compounds (VOCs) was also higher in the ultrasound-treated group compared to the control [11]. High-pressure homogenization (HPH) is a food processing technique that refines and uniformly disperses particles or droplets by forcing liquid materials through a narrow valve under extreme pressure, which subjects them to intense shear, impact, and cavitation. In a related approach, Feng et al. [12] demonstrated that homogenization combined with ultrasonic treatment (UST) enhanced the antioxidant activity of chicken soup, promoted the formation of volatile flavor compounds (VFCs), and increased the content of FAAs and 5′-nucleotides.

In addition, Ye et al. [13] investigated the effects of ultrasound-assisted combined with high-pressure processing (UHBBS) on flavor compounds in beef bone broth. It was demonstrated that UHBBS treatment significantly enhanced the umami intensity, with the total nucleotide content increased by 34.18% and the total FAA content increased by 150.62%. Roobab et al. [14] studied the impact of combined ultrasound and pulsed electric field treatments on the quality of chicken breast. The results indicated that ultrasonic pretreatment combined with different intensities of PEF significantly reduced cooking loss (by up to 28.78%) and induced a tenderizing effect through disruption of muscle fiber structure, leading to notable decreases in hardness, chewiness, and gumminess. Kou et al. [15] compared the physicochemical and structural properties of pork bone soup prepared under high-pressure (170 kPa) and atmospheric (100 kPa) stewing conditions. It was found that atmospheric stewing promoted the emulsification of proteins and lipids, resulting in a milky and uniform soup, whereas high-pressure stewing significantly improved the release efficiency of nutrients but caused excessive protein unfolding and aggregation. Compared with other analytical techniques, gas chromatography–mass spectrometry (GC–MS) offers distinct advantages in flavor analysis [16]. It provides exceptionally high detection sensitivity, and the use of established standard spectral libraries allows for the rapid identification of unknown metabolites by comparing mass spectral fragmentation patterns [17].

While the Wuliang Mountain black-boned chicken, a local breed from Yunnan, has been studied primarily in animal science, research on its food product development and flavor characteristics is lacking. To address this gap, chicken soups were prepared using three processing methods: conventional atmospheric stewing, ultrasonic-assisted low-temperature stewing, and HPH. A comprehensive assessment of the soups was conducted based on sensory quality, FAAs, free fatty acids (FFAs), and volatile flavor compounds (VFCs), utilizing analytical methodologies including an amino acid analyzer and GC–MS. This work aims to provide a scientific foundation for the resource utilization, development, and conservation of this local chicken breed.

## 2 Materials and methods

### 2.1 Materials and chemicals

Fifteen 365-day-old Wuliang Mountain black-boned hens from the same batch and raised under identical conditions were obtained from Dali Shanyu Agriculture Co., Ltd. (Dali, China). The following reagents were used: sucrose ester was purchased from Henan Zhongchen Bio-Technology Co., Ltd. (Zhengzhou, China); 0.1 mol/L HCl was supplied by Tianjin Damao Chemical Reagent Factory (Tianjin, China); acidic trisodium citrate aqueous solution was acquired from Shanghai Macklin Biochemical Co., Ltd. (Shanghai, China); alkaline trisodium citrate aqueous solution was obtained from Shanghai Sinopharm Chemical Reagent Co., Ltd. (Shanghai, China); sodium hydroxide was provided by Tianjin Fengchuan Chemical Reagent Co., Ltd. (Tianjin, China); and ninhydrin-methanol solution was sourced from Sinopharm Chemical Reagent Co., Ltd. (Shanghai, China).

The sample preparation procedure was conducted as follows: carcasses from 15 Wuliang Mountain black-boned chickens were cut into pieces, thoroughly mixed, and divided into three equal groups (each containing meat from 5 chickens) using stratified random sampling, designated as the control group (C group), the ultrasound-treated group (U group), and the HPH group (H group). The procedure for C group, based on parameters optimized in preliminary trials (the relevant findings have been accepted by the Chinese core journal *China Condiment*), involved cutting 100 g of chicken pieces, blanching them in boiling water (100 °C) for 1 min, placing the pieces in a beaker with 200 ml of water and 1.7 g of salt, sealing the beaker with plastic film, and stewing it in a constant-temperature water bath (Zhengrong Experimental Instrument Co., Ltd., Changzhou, China) at 100 °C for 2 h. After stewing, the samples were cooled to room temperature for sensory evaluation and various measurements, and the remainder was stored at −80 °C for further use. The procedure for U group was adapted from the method of Yue et al. [11]: the processing steps, ingredient quantities, and stewing time were identical to C group, except that the beaker was placed inside an ultrasonic cleaner (Jiemeng Technology Co., Ltd., Shenzhen, China) for assisted cooking at a power of 800 W and a temperature of 80 °C. The procedure for H group was modified from the method of Colle et al. [18]: the basic stewing process was the same as Group C, but after 2 h of stewing, 0.7% sucrose ester was added to the chicken broth and stirred until completely dissolved. The broth was then treated with a homogenizer at 12,800 × g for 2 min at room temperature (Instrument Laboratory Technology Co., Ltd., Guangzhou, China) and processed using a high-pressure homogenizer (Litu Machinery Equipment Engineering Co., Ltd., Shanghai, China) for 7 cycles at a pressure of 200 bar and room temperature.

### 2.2 Sensory evaluation

Sensory analysis was performed in a standardized sensory evaluation room, with each sample assessed in triplicate. A trained sensory panel consisting of 20 food science graduate students (10 males and 10 females, aged 20–30 years) evaluated the chicken broth. All participants provided informed consent and had no history of taste or olfactory disorders, including basic impairments in saltiness or bitterness perception. The broth was scored on a 100-point scale based on color, surface oil, aroma, and taste, following the criteria outlined in Table S1.

### 2.3 Determination of free amino acids

#### 2.3.1 Experimental methods

The testing method was adapted from Yuan et al. [19] with modifications. Briefly, samples were ground into a powder using liquid nitrogen, and exactly 1000 mg was accurately weighed and diluted to 50 mL with 0.1 mol/L HCl in a volumetric flask. The mixture was subjected to ultrasonication for 30 min, vortex mixing, a second 30-min ultrasonication, and finally centrifugation at 12,000 rpm for 10 min. The resulting supernatant was filtered through a 0.22 μm membrane to obtain the test solution. Analysis was performed on an amino acid analyzer (Secam Scientific Instruments Co., Ltd., Munich, Germany) under the following conditions: a cation exchange column (4.6 × 150 mm) maintained at 130 ℃; an injection volume of 20 μL; and detection at 570 nm and 440 nm using a fixed dual-wavelength visible detector. The mobile phase system consisted of acidic aqueous trisodium citrate (A), alkaline aqueous trisodium citrate (B), with sodium hydroxide as the regenerant, water as the wash solution, and ninhydrin in methanol as the reaction reagent.

#### 2.3.2 Taste activity value calculation

Taste activity value (TAV) is defined as the ratio of the relative concentration of taste substance to its taste threshold, which reflects the contribution of a taste substance to the overall taste [20]. The formula is as follows:

$$\mathrm{TAV}=\frac{\text{C}}{\text{T}}$$

Where C represents the absolute concentration of taste substance (mg/kg); T represents the threshold of taste substance (mg/kg).

### 2.4 Determination of free fatty acids by GC–MS

The testing method was adapted from Hoving et al. [21] with modifications. Gas chromatography was performed on a Thermo Trace 1300 system (Thermo Fisher Scientific, Waltham, MA, USA) equipped with a TG-FAME capillary column (50 m × 0.25 mm i.d. × 0.20 μm). A 1 μL sample was injected in split mode (8:1 ratio). The injector, ion source, and transfer line temperatures were set to 250 ℃, 300 ℃, and 280 ℃, respectively. The oven temperature program was as follows: held at 80 ℃ for 1 min, increased to 160 ℃ at 20 ℃/min and held for 1.5 min, then raised to 196 ℃ at 3 ℃/min and held for 8.5 min, and finally increased to 250 ℃ at 20 ℃/min with a 3 min hold. Helium was used as the carrier gas at a constant flow rate of 0.63 mL/min. Mass spectrometric detection was carried out using a Thermo TSQ 9000 instrument (Thermo Fisher Scientific) with an electron ionization (EI) source operated at 70 eV in selected ion monitoring (SIM) mode.

### 2.5 Determination of volatile flavor compounds by GC–MS

#### 2.5.1 Sample preparation

The fiber (Anpu Experimental Technology Co., Ltd., Shanghai, China) was initially conditioned at 250 ℃ for 15 min. Subsequently, a 5 mL aliquot of chicken soup was transferred to a 15 mL headspace vial, followed by the addition of 1.5 μL o-dichlorobenzene as an internal standard. The vial was equilibrated for 10 min in a water bath maintained at 55 ℃. The conditioned fiber was then inserted into the vial headspace (CNW Technologies GmbH, Dusseldorf, Germany) for a 30-min adsorption period. Finally, the fiber was introduced directly into the GC inlet for thermal desorption over 10 min before being removed.

#### 2.5.2 GC–MS analysis

The testing conditions were adapted from Shen et al. [22]. Gas chromatography (Agilent Technologies Inc., Santa Clara, CA, USA) was performed using an HP-INNOWAX quartz capillary column (60 m × 0.25 mm × 0.25 μm, polar) with a split ratio of 5:1. The injector temperature was maintained at 250 ℃, and helium was used as the carrier gas at a flow rate of 10 mL/min. The oven temperature program was set as follows: initial temperature of 40 ℃ held for 3 min, increased to 90 ℃ at 5 ℃/min and held for 10 min, then raised to 230 ℃ at 10 ℃/min with a final hold of 15 min. The solvent delay was 5 min. For mass spectrometry (Agilent Technologies Inc.), electron ionization (EI) at 70 eV was used. The ion source, quadrupole, and interface temperatures were set to 230 ℃, 150 ℃, and 250 ℃, respectively. The mass scan range was 50–550 amu in full scan mode, with a solvent delay of 7 min.

#### 2.5.3 Qualitative analysis of volatile flavor compounds

The identification of VFCs was based on mass spectral library matching and comparison of retention indices (RIs). The Chemical Abstracts Service (CAS) numbers and documented RIs for the polar capillary column were retrieved from the NIST standard spectral database (NIST Chemistry WebBook, SRD 69). A compound was considered identified if the deviation between its calculated RI and the documented reference value was within 50. The retention indices for unknown compounds were determined by analyzing a homologous series of n-alkanes (C7–C30) under the same GC–MS conditions and were calculated from their measured retention times using the following formula:

$$\mathrm{RIs}=100\times Z+100\times \frac{\left(RT_{x}-RT_{z}\right)}{\left(RT_{z+1}-RT_{z}\right)}$$

Where:

RTX: Retention time/(min) of the volatile component;

RTZ: Retention time/(min) of the n-alkane with the same number of carbon atoms as the volatile component;

Z: Carbon atom number of a specific n-alkane;

After chromatographic column analysis, the peak of component X lies exactly between the n-alkanes with carbon atom numbers Z and Z + 1.

#### 2.5.4 Quantitative analysis of volatile flavor compounds

Simple quantification was performed using the internal standard method, with o-dichlorobenzene at a concentration of 100 ppm selected as the internal standard. The relative content (Wi) of each volatile aroma component was calculated accordingly. The formula is as follows:

$$\text{Wi}=\frac{C_{S}\times V_{S}}{m}\times \frac{\mathrm{APi}}{\mathrm{APs}}$$

Where:

Wi: Mass concentration of the volatile component to be measured/(μg/L);

Cs: Concentration of the internal standard/(μg/L);

Vs: Volume of the internal standard added/(μL);

M: Mass of the sample weighed/(g);

APi: Peak area of the volatile component (Area Pct);

APs: Peak area of the internal standard (Area Pct).

#### 2.5.5 Odor activity value calculation

The odor activity value (OAV) is mainly used to evaluate the contribution of individual volatile compounds in food to the overall aroma [23]. The OAV for each VFCs was calculated by referencing its odor threshold in water, which was obtained by searching the detection threshold database [24]. The formula is as follows:

$$\mathrm{OAV}=\frac{\text{A1}}{A2}$$

Where:

A1: Concentration of a specific flavor compound in the sample (μg/L).

A2: Odor threshold of the compound (μg/L).

### 2.6 Data statistics and analysis

Data analysis was performed using SPSS 26.0 for statistical analysis. Origin 2021 was used for graphing, data normalization, principal component analysis (PCA), and Pearson correlation analysis. Orthogonal partial least squares-discriminant analysis (OPLS-DA) was conducted using SIMCA 14.1, and the variable importance in projection (VIP) values were calculated.

## 3 Results and discussion

### 3.1 Analysis of sensory evaluation

**Fig. 1:**
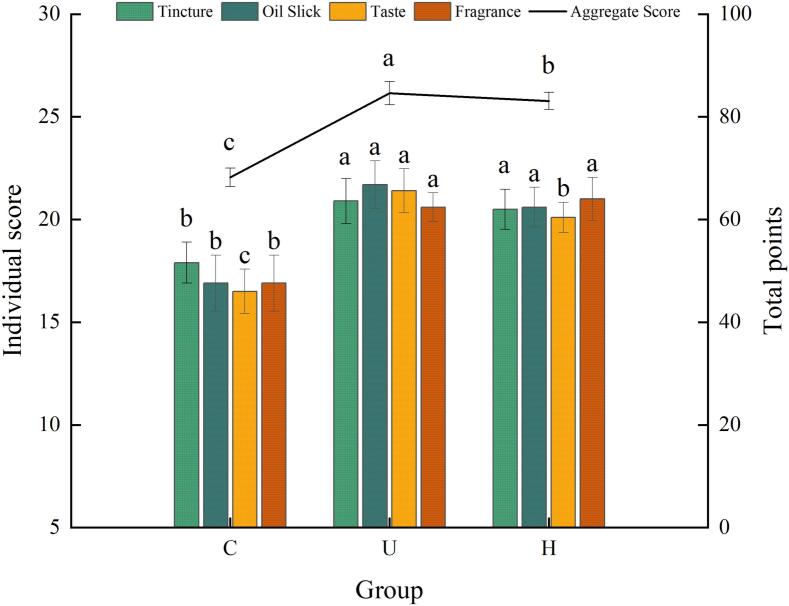
Sensory scores of chicken soup samples. The x-axis represents the group, and the y-axis represents the score. Each color corresponds to a different scoring metric, and a horizontal reference line indicates the total score.

The sensory evaluation results are presented in Fig. 1. The overall sensory scores decreased in the order U > H > C, with significant differences (*p* < 0.05) observed among the groups. This result demonstrates that physical processing technologies (ultrasound and HPH) yielded chicken soups with superior overall sensory quality compared to conventional stewing. For the individual attributes of color, surface oil, aroma, and taste, the highest scores were consistently obtained by the U group, followed by the H and C groups. With the exception of the aroma attribute, for which all three groups differed significantly (*p* < 0.05), no significant difference was observed between the U and H groups (*p* > 0.05), although both differed significantly from the C group (*p* < 0.05). The superior overall and individual attribute scores of the U group are likely attributable to the ultrasonic mechanism. This mechanism is driven by acoustic cavitation, wherein cavitation bubbles within the liquid medium are generated, grow, and violently collapse under ultrasonic excitation [25]. This cavitation effect efficiently disrupts ingredient cell walls and membranes, facilitating the release of flavor compounds—including amino acids, peptides, nucleotides, sugars, VOCs, and lipids [26]—thereby enriching the soup's flavor and aroma profile. Furthermore, the collapse of these bubbles generates intense shock waves and microjets that disrupt large fat globules [27], resulting in a finer and more uniform dispersion of fat droplets. This improvement stabilizes the oil distribution within the soup, thereby reducing surface oil and enhancing both its texture and appearance. In the H group, HPH effectively reduced the disperse phase to micron-scale particles, which significantly improved emulsion stability [28], decreased fat globule size, and promoted a more uniform dispersion of flavor compounds. These changes consequently enhanced the soup's texture and flavor release profile. In contrast, the conventional stewing method used in the C group promoted fat aggregation into large oil droplets. These droplets migrated to the surface, forming a distinct oily layer that imparted a greasy sensation. The large size of the fat globules also hindered effective flavor release [29].

### 3.2 Analysis of free amino acids

**Fig. 2:**
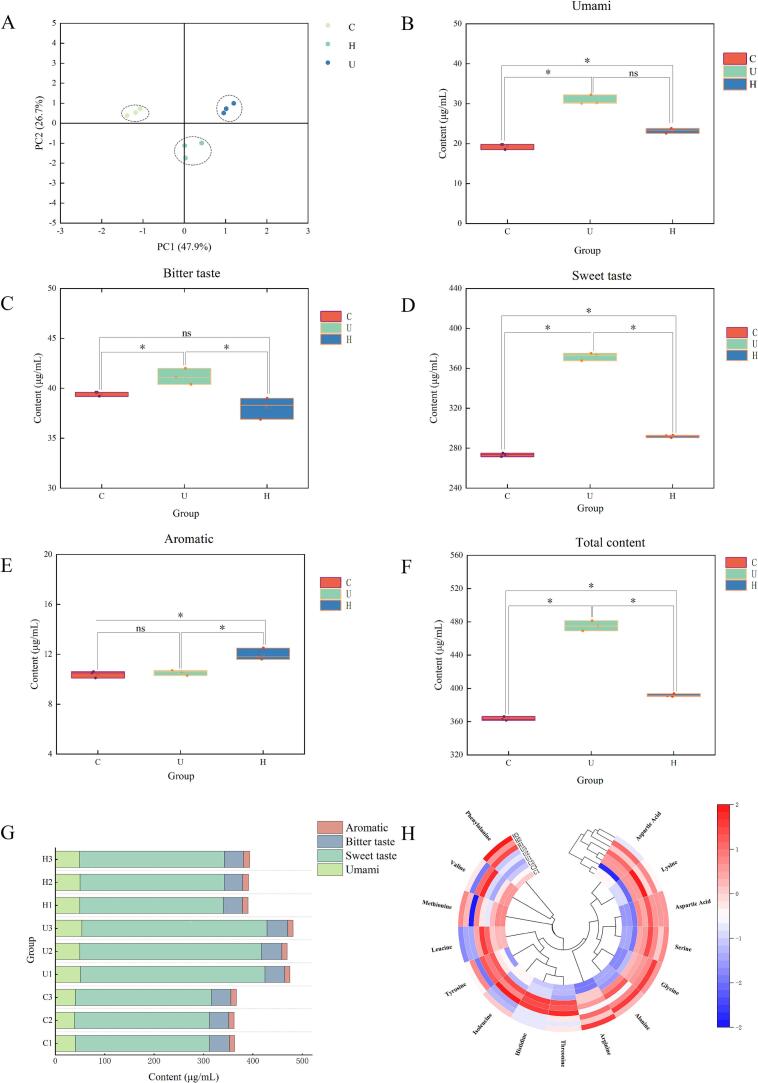
Free amino acid detection results of three chicken broth samples. (A) PCA score plot, (B) Box plot of umami amino acids, (C) Box plot of bitter amino acids, (D) Box plot of sweet amino acids, (E) Box plot of aromatic amino acids, (F) Box plot of total amino acid content, (G) Bar chart of amino acid content, (H) Cluster heatmap of amino acids in the three chicken broth samples. “*” *p* < 0.05, “ns” *p* > 0.05. In Figure (A), the x- and y-axes represent the on-chart distances, while PC1 and PC2 indicate the proportion of variance explained by the principal components. For Figures (B) through (F), the x-axis denotes the experimental groups, and the y-axis shows the concentration of the substance (in µg/mL). In Figure (G), substance concentration (µg/mL) is plotted on the x-axis, groups are shown on the y-axis, and different colors represent distinct substance categories. Finally, in Figure (H), each colored block corresponds to one substance, with notes at the start of each block indicating the group. Color intensity follows a gradient from blue (low concentration) to red (high concentration).

FAAs, important as nonprotein nitrogen compounds, serve as key flavor components and precursors in chicken meat [30]. The FAA contents for the three sample groups are shown in Table S2 and Fig. 2. PCA revealed clear separation among the groups (Fig. 2A), indicating significant inter-group differences, while the close clustering of replicate samples within each group confirmed high experimental repeatability and reliability. Based on their taste characteristics, the FAAs were categorized as umami, bitter, sweet, or aromatic. The contents of umami amino acids were significantly higher in the U and H groups than in the C group (*p* < 0.05, Fig. 2B). Specifically, the concentrations of glutamic acid (32.03 and 31.03 mg/L in the U and H groups, respectively) and lysine (11.77 and 11.20 mg/L) in these groups were significantly elevated compared to the C group (*p* < 0.05). For bitter amino acids (Fig. 2C), the U group exhibited a significantly higher content than both the C and H groups (*p* < 0.05). Regarding sweet amino acids (Fig. 2D), a significant decreasing trend was observed in the order: U group > H group > C group (*p* < 0.05). Notably, the histidine content in the U group (314.23 mg/L) was 1.35 times and 1.30 times greater than in the C group (233.40 mg/L) and H group (240.90 mg/L), respectively. The content of aromatic amino acids was significantly higher in the H group than in the other groups (*p* < 0.05, Fig. 2E). In the comparison of total FAA content (Fig. 2F), the U group was found to be significantly higher than the H and C groups (*p* < 0.05), an increase which was principally due to the elevated level of histidine within the sweet amino acid category. In summary, UST significantly enhanced the content of FAAs, particularly key taste-active compounds such as glutamic acid, lysine, and histidine. In contrast, HPH increased the levels of other taste-related amino acids, including phenylalanine and glycine.

A cluster heatmap was constructed to further analyze the differences in FAA profiles among the groups (Fig. 2G). Higher concentrations of aspartic acid, glutamic acid, lysine, threonine, serine, glycine, alanine, histidine, isoleucine, leucine, arginine, and tyrosine were observed in the U group. Similarly, the H group showed elevated levels of methionine, phenylalanine, arginine, alanine, glycine, serine, glutamic acid, and aspartic acid. These results indicate that both ultrasonic and HPH treatments effectively increased the concentrations of various taste-active amino acids, which is expected to contribute to the flavor enhancement of the chicken broth. The results of the TAV analysis are summarized in Table S3. Among all amino acids analyzed, only histidine exhibited a TAV greater than 1 in all three groups, with values of 1.167 (C group), 1.571 (U group), and 1.205 (H group), as shown in Table S3. Furthermore, histidine was found to have the highest absolute concentration among all amino acids (233.40 mg/L in C group, 314.23 mg/L in U group, and 240.90 mg/L in H group, from Table S2 and Fig. 2). Consequently, based on its superior concentration and confirmed taste impact (TAV > 1), histidine was identified as a key taste-active amino acid in the chicken broth.

Histidine, an essential amino acid for humans, contributes to umami taste, especially when synergistically combined with nucleotides like inosine monophosphate and guanosine monophosphate, leading to a significant enhancement of this flavor [31]. Furthermore, histidine acts as a precursor in the Maillard reaction. During cooking, it reacts with reducing sugars to produce various volatile compounds that are important for the characteristic flavor of meat [32]. In the present study, UST significantly increased the histidine content. This effect is likely attributable to the high-pressure shockwaves and shear forces generated by ultrasonic cavitation, which disrupt the tertiary and quaternary structures of myofibrillar proteins. This structural disruption leads to protein unfolding, thereby exposing buried histidine residues and rendering cleavage sites adjacent to histidine more accessible to endogenous proteases (e.g., cathepsins and calpains), consequently promoting the release of free histidine [33]. In conclusion, UST effectively enhanced the content of flavor-contributing amino acids, leading to an improved taste profile of the chicken soup. Although HPH also increased the content of these amino acids to a certain extent, its effect was less pronounced than that of UST.

### 3.3 Analysis of free fatty acids

Fatty acids, which are the fundamental structural units of lipids such as triglycerides and phospholipids [34], play a significant role in flavor formation. For example, unsaturated fatty acids (e.g., linoleic and linolenic acid) can undergo autoxidation, photooxidation, or enzymatic oxidation to form hydroperoxides. These unstable intermediates subsequently decompose into volatile compounds, including aldehydes, ketones, and alcohols [35]. Fatty acids are categorized by their degree of unsaturation into saturated (SFA), monounsaturated (MUFA), and polyunsaturated (PUFA) fatty acids. The composition and content of FFAs in the chicken soups processed with the three different treatments are summarized in Table 1. In total, 47 FFAs were detected in the C and U groups, consisting of 14 SFA, 21 MUFA, and 12 PUFA. In contrast, 49 FFAs were identified in the H group, which included 16 SFA, 21 MUFA, and 12 PUFA.

**Table 1:** Free fatty acid content and composition of chicken soup samples.

Types of free fatty acid	Name	Free fatty acid composition and content(μg/mL)		
		C group	U group	H group
C6:0	caproic acid	−	−	0.06 ± 0.10
C8:0	caprylic acid	−	−	0.76 ± 0.26
C10:0	capric acid	＜0.01	＜0.01	0.74 ± 0.03
C11:0	undecanoic acid	＜0.01	＜0.01	0.02 ± 0.008
C12:0	lauric acid	0.04 ± 0.01^b^	0.05 ± 0.02^b^	7.55 ± 0.40^a^
C13:0	tridecanoic acid	0.01 ± 0.002^b^	0.01 ± 0.003^b^	0.05 ± 0.006^a^
C14:0	myristic acid	0.28 ± 0.14^b^	0.27 ± 0.11^b^	20.75 ± 1.36^a^
C14:1T	myristic acid	0.30 ± 0.06^a^	0.32 ± 0.02^a^	0.31 ± 0.01^a^
C14:1	myristoleic acid	0.20 ± 0.06^b^	0.19 ± 0.03^b^	1.51 ± 0.09^a^
C15:0	pentadecanoic acid	0.09 ± 0.01^b^	0.09 ± 0.01^b^	1.69 ± 0.13^a^
C15:1T	(E)-pentadecenoic acid	0.34 ± 0.08^a^	0.32 ± 0.05^a^	0.33 ± 0.03^a^
C15:1	*cis*-pentadecenoic acid	0.13 ± 0.03^a^	0.12 ± 0.02^a^	0.17 ± 0.01^a^
C16:0	palmitic acid	8.77 ± 4.55^b^	8.09 ± 3.38^b^	189.41 ± 4.93^a^
C16:1T	(E)-hexadec-9-enoic acid	0.13 ± 0.04^b^	0.15 ± 0.03^bb^	0.45 ± 0.03^a^
C16:1	palmitoleic acid	1.14 ± 0.66^b^	0.80 ± 0.31^b^	52.52 ± 5.04^a^
C17:0	heptadecanoic acid	0.16 ± 0.04^b^	0.16 ± 0.03^b^	3.78 ± 0.41^a^
C17:1T	(E)-heptadecenoic acid	0.25 ± 0.05^b^	0.24 ± 0.04^b^	1.50 ± 0.10^a^
C17:1	*cis*-10-heptadecenoic acid	0.31 ± 0.05^b^	0.31 ± 0.05^b^	0.91 ± 0.14^a^
C18:0	stearic acid	3.56 ± 1.04^b^	3.23 ± 1.16^b^	58.67 ± 1.25^a^
C18:1N9T	elaidic acid	0.27 ± 0.02^c^	0.34 ± 0.02^b^	2.69 ± 0.01^a^
C18:1N7T	(E)-octadec-9-enoic acid	0.05 ± 0.03^b^	0.07 ± 0.02^b^	0.56 ± 0.04^a^
C18:1N12	*cis*-6-octadecenoic acid	2.04 ± 1.32^b^	3.89 ± 2.55^b^	33.53 ± 17.78^a^
C18:1N9C	oleic acid	11.33 ± 7.21^b^	9.76 ± 4.61^b^	473.07 ± 30.57^a^
C18:1N7	*cis*-6-ooctadecenoic acid	0.72 ± 0.49^b^	0.60 ± 0.19^b^	24.07 ± 2.92^a^
C18:2N6T	(9E,12E)-octadeca-9,12-dienoic acid	0.03 ± 0.02^b^	0.04 ± 0.02^b^	0.19 ± 0.03^a^
C19:1N9T	(E)-nonadecenoic acid	0.10 ± 0.02^b^	0.09 ± 0.01^b^	0.48 ± 0.02^a^
C18:2N6	Linoleic acid (LA)	6.95 ± 4.44^b^	5.81 ± 2.99^b^	330.89 ± 27.00^a^
C20:0	arachidic acid	0.14 ± 0.02^b^	0.14 ± 0.02^b^	5.25 ± 0.46^a^
C18:3N6	γ-Linolenic acid (GLA)	0.11 ± 0.03^b^	0.11 ± 0.03^b^	1.53 ± 0.06^a^
C20:1T	(E)-eicosenoic acid	0.08 ± 0.06^b^	0.10 ± 0.03^b^	0.32 ± 0.04^a^
C20:1	(Z)-11-eicosenoic acid	0.21 ± 0.09^b^	0.21 ± 0.07^b^	4.60 ± 0.43^a^
C18:3N3	α-Linolenic acid (ALA)	0.30 ± 0.14^b^	0.27 ± 0.09^b^	8.72 ± 0.57^a^
C21:0	heneicosanoic acid	0.08 ± 0.01^b^	0.08 ± 0.01^b^	0.22 ± 0.02^a^
C20:2	eicosadienoic acid	0.06 ± 0.04^b^	0.08 ± 0.05^b^	2.26 ± 0.22^a^
C22:0	behenic acid	0.06 ± 0.01^b^	0.06 ± 0.01^b^	0.84 ± 0.09^a^
C20:3N6	Dihomo-γ-linolenic acid (DGLA)	0.10 ± 0.04^b^	0.10 ± 0.03^b^	1.69 ± 0.12^a^
C22:1N9T	(E)-docos-13-enoic acid	0.15 ± 0.10^b^	0.13 ± 0.05^b^	0.33 ± 0.11^a^
C22:1N9	erucic acid	0.11 ± 0.04^b^	0.13 ± 0.02^b^	0.39 ± 0.05^a^
C20:3N3	eicosatrienoic acid	0.08 ± 0.01^b^	0.08 ± 0.02^b^	0.25 ± 0.03^a^
C20:4N6	Arachidonic acid (ARA)	0.20 ± 0.04^b^	0.16 ± 0.05^b^	4.31 ± 0.26^a^
C23:0	tricosanoic acid	0.06 ± 0.005^b^	0.07 ± 0.008^b^	0.22 ± 0.01^a^
C22:2	docosadienoic acid	0.04 ± 0.03^b^	0.06 ± 0.03^b^	0.20 ± 0.05^a^
C20:5N3	Eicosapentaenoic acid (EPA)	0.07 ± 0.01^b^	0.08 ± 0.02^b^	0.25 ± 0.02^a^
C24:0	lignoceric acid	0.07 ± 0.10^b^	0.07 ± 0.01^b^	0.74 ± 0.07^a^
C24:1	nervonic acid	0.18 ± 0.07^b^	0.18 ± 0.05^b^	0.75 ± 0.14^a^
C22:4	adrenic acid	0.13 ± 0.03^b^	0.13 ± 0.03^b^	1.67 ± 0.12^a^
C22:5N6	Docosapentaenoic acid (ω-6)	0.10 ± 0.02^b^	0.11 ± 0.03^b^	0.35 ± 0.04^a^
C22:5N3	Docosapentaenoic acid (DPA)	0.08 ± 0.01^b^	0.08 ± 0.02^b^	0.58 ± 0.06^a^
C22:6N3	Docosahexaenoic acid (DHA)	0.06 ± 0.02^b^	0.06 ± 0.02^b^	0.34 ± 0.03^a^
SFA	Saturated fatty acid	13.40 ± 5.94^b^	12.39 ± 4.80^b^	286.89 ± 3.32^a^
MUFA	Monounsaturated fatty acid	1.18 ± 0.38^b^	1.25 ± 0.32^b^	13.76 ± 1.09^a^
PUFA	Polyunsaturated fatty acid	17.87 ± 10.08^b^	17.62 ± 7.72^b^	608.23 ± 24.51^a^
TFFA	Total free fatty acid	32.45 ± 13.35^b^	31.25 ± 10.45^b^	908.88 ± 18.17^a^

Note: “-” indicates not detected.

**Fig. 3:**
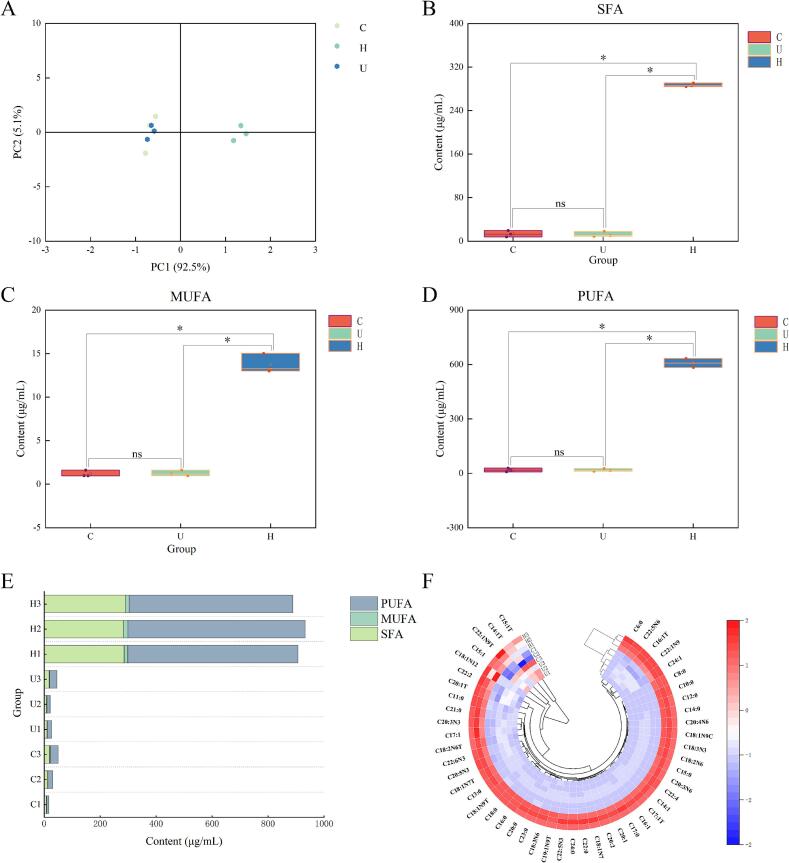
Free fatty acid detection results of three chicken broth samples. (A) PCA score plot, (B) Box plot of saturated fatty acids, (C) Box plot of monounsaturated fatty acids, (D) Box plot of polyunsaturated fatty acids, (E) Bar chart of total free fatty acid content, (F) Cluster heatmap of fatty acids in the three chicken broth samples. “*” *p* < 0.05, “ns” *p* > 0.05. In Figure (A), the x- and y-axes correspond to the on-chart distance, with PC1 and PC2 denoting the proportion of variance explained by the principal components. In Figures (B) through (D), the x-axis represents the experimental groups, and the y-axis shows the concentration of the substance (µg/mL). Figure (E) displays substance concentration (µg/mL) on the x-axis and groups on the y-axis, with distinct colors representing different substance categories. In Figure (F), each colored bar corresponds to a substance. The groups are labeled in a note at the beginning of each bar, and concentration is encoded on a color gradient from blue (low) to red (high).

PCA revealed a clear separation in the fatty acid profiles among the groups (Fig. 3A). The sample points for the C and U groups clustered closely with considerable overlap, indicating a high similarity in their overall fatty acid composition. In contrast, the H group was clearly separated, suggesting a distinct profile. Specifically, the contents of SFA, MUFA, and PUFA were all significantly higher in the H group than in the U and C groups (*p* < 0.05; Fig. 3B–3E), while no significant differences were detected between the C and U groups (*p* > 0.05). To visualize the inter-group differences in fatty acid content, cluster heatmap analysis was performed (Fig. 3F). The clear clustering of samples within their respective groups indicated good experimental reproducibility and reliability. Furthermore, the H group demonstrated markedly higher levels of individual FFAs compared to the U and C groups, confirming that HPH effectively increased the FFA content. Specifically, the contents of palmitic acid, oleic acid, and linoleic acid in the H group (189.41, 473.03, and 330.39 μg/mL, respectively) were 21.6, 41.8, and 47.5 times greater than those in the C group. Consequently, the total fatty acid content in the H group (621.99 μg/mL) was 32.65 times that of the C group.

Palmitic acid, a SFA, exhibits greater stability against oxidation and is involved in cellular membrane formation and energy metabolism [36]. However, excessive intake has been linked to an increased risk of cardiovascular diseases, as it may elevate levels of low-density lipoprotein cholesterol (LDL-C) [37]. It should be noted, however, that its health impact is considered dependent on the ratio of other dietary fatty acids and individual metabolic conditions [38]. Although palmitic acid itself does not contribute significant direct flavor, as a structural lipid it confers desirable textural properties to foods, such as creaminess, smoothness, and richness [39]. Furthermore, by serving as a lipophilic matrix, it can dissolve and retain numerous lipid-soluble flavor compounds. This contributes to the stabilization and prolonged release of these flavor substances [40], thereby enhancing the overall flavor perception of foods. Separately, oleic acid, a MUFA, has been shown to reduce blood cholesterol levels, improve cell membrane permeability, and aid in the prevention of myocardial blockage and arteriosclerosis [41]. It possesses a mild flavor but serves as a crucial flavor carrier in foods, facilitating the delivery of lipid-soluble flavor compounds and aiding in the absorption of fat-soluble vitamins [42]. Linoleic acid, an essential PUFA for humans, performs vital physiological functions including prostaglandin synthesis, cellular membrane construction, and the maintenance of skin, cardiovascular, and immune health [43], [44], [45], [46].

The oxidation of linoleic acid yields a series of volatile compounds, including aldehydes, ketones, alcohols, and acids. For instance, 2,4-decadienal—an oxidative product of linoleic acid—imparts a characteristic deep-fried, fatty flavor and is recognized as a key aroma compound in numerous foods [47]. Moreover, linoleic acid and its derivatives can serve as precursors for specific flavor substances; during meat processing, for example, the oxidation of linoleic acid interacts synergistically with the Maillard reaction to generate complex flavor compounds [48]. The increase in fatty acids resulting from HPH is likely due to the disruption of cellular structures and adipose tissues, which facilitates the release of encapsulated or bound lipid components. It is crucial to note that fatty acids are not generated de novo during this process; rather, homogenization releases those originally embedded within adipose tissues or cellular matrices, thereby markedly increasing the levels of extractable FFAs. Additionally, lipases naturally present in the chicken soup may hydrolyze triglycerides, contributing further to the pool of FFAs [12]. The activity of such enzymes can itself be influenced by ultra-high pressure treatment, which may either activate or inactivate them depending on the specific enzyme and processing conditions [49]. Meanwhile, research has also indicated that HPH can improve fat digestibility and bioaccessibility. It has been shown to enhance lipid digestion rates, thereby indirectly increasing the bioavailability of fatty acids in vivo, even when the total lipid content in the food matrix remains unchanged [50]. In summary, this study demonstrates that HPH significantly increases the content of FFAs—particularly palmitic, oleic, and linoleic acids—effectively enhancing both the nutritional value and the flavor profile of the chicken soup.

### 3.4 Analysis of volatile flavor compounds by GC–MS

#### 3.4.1 Analysis of composition and content of volatile flavor compounds

**Fig. 4:**
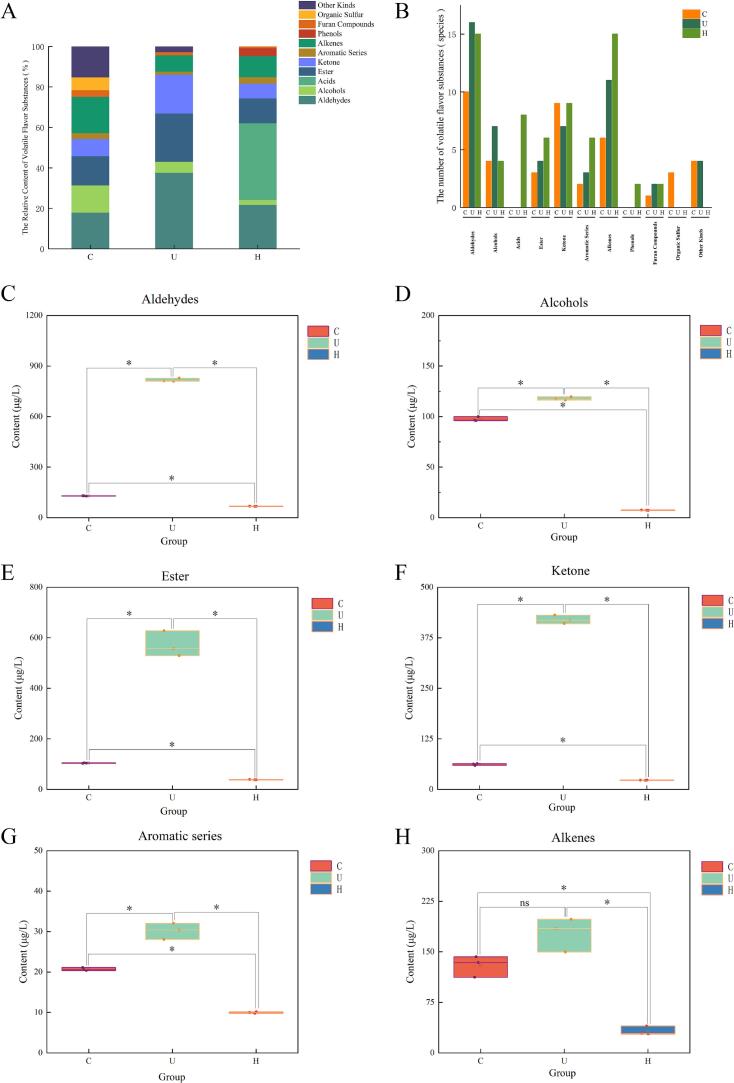
Determination results of volatile flavor compounds in different chicken broth samples. (A) Percentage bar chart of compounds, (B) Category bar chart of compounds, (C) Box plot of aldehydes, (D) Box plot of alcohols, (E) Box plot of esters, (F) Box plot of ketones, (G) Box plot of aromatic compounds, (H) Box plot of alkenes. “*” *p* < 0.05, “ns” *p* > 0.05. In Figure (A), the x-axis represents the experimental groups and the y-axis shows the substance concentration (µg/mL), with each substance represented by a distinct colored bar. Figure (B) displays substance categories on the x-axis and concentration (µg/mL) on the y-axis, where different colors correspond to different experimental groups. For Figures (C) through (H), the x-axis indicates the experimental groups, and the y-axis indicates the substance concentration (µg/mL).

Analysis of the VFCs via GC–MS detected a total of 107 compounds across the three chicken broth types (Table 2). The relative abundance of 11 flavor categories is shown in Fig. 4A. The U group exhibited higher proportions of aldehydes (37.57%), esters (23.81%), and ketones (19.32%), whereas the H group was characterized by a high relative content of acids (38.01%). In contrast, the C group showed elevated proportions of alcohols (13.48%), alkenes (17.93%), organosulfur compounds (6.34%), and other compounds (15.3%) compared to the other two groups. As illustrated in Fig. 4B, the H group contained a significantly greater number of volatile compound types (67) than both the U group (54) and C group (42) (*p* < 0.05). Differential analysis of the compound categories common to all groups (Fig. 4C–4H) revealed that the U group had significantly higher contents of aldehydes, alcohols, esters, ketones, aromatic compounds, and alkenes than the C and H groups (*p* < 0.05), indicating that UST markedly increased these substances. Specifically, the concentrations in the U group—aldehydes (816.33 μg/L), alcohols (117.82 μg/L), esters (571.35 μg/L), ketones (419.86 μg/L), aromatic compounds (30.17 μg/L), and furans (33.29 μg/L)—were significantly greater than in the other groups (*p* < 0.05). In contrast, the H group showed significant increases only in acids (118.23 μg/L) and phenols (12.31 μg/L), while its contents in other categories were generally lower than in the C group. The C group exhibited higher levels of organosulfur compounds (45.85 μg/L) and other compounds (110.61 μg/L). In summary, UST significantly enhanced both the variety and content of key volatile flavor compounds, whereas HPH primarily increased compound diversity but notably elevated only acids and phenols.

**Table 2:** The results of composition and content of volatile flavor compounds in chicken soup.

Class	CAS	Name	Volatile flavor compounds contents(μg/L)		
			C group	U group	H group
Aldehydes	66–25-1	hexanal	37.42 ± 1.05^b^	287.10 ± 7.13^a^	6.22 ± 0.02^c^
	18829–55-5	(E)-2-heptenal	8.97 ± 0.02^b^	50.82 ± 0.14^a^	6.36 ± 0.12^c^
	124–19-6	nonanal	12.69 ± 0.70^b^	112.01 ± 0.58^a^	4.09 ± 0.10^c^
	2548–87-0	(E)-2-octenal	23.92 ± 0.29	49.06 ± 1.18	−
	112–31-2	decanal	7.93 ± 0.81^c^	9.30 ± 0.19^b^	10.68 ± 0.32^a^
	100–52-7	benzaldehyde	19.76 ± 0.38^a^	20.16 ± 0.02^a^	4.90 ± 0.63^b^
	5910–87-2	(E,E)-2,4-nonadienal	2.51 ± 0.05	21.11 ± 0.09	−
	51534–36-2	(2E)-2-tetradecenal	6.13 ± 0.02	−	1.65 ± 0.05
	587–4-2	3-chlorobenzaldehyde	3.85 ± 0.10	−	−
	25152–83-4	(E,Z)-2,4-decadienal	5.98 ± 0.05	0.11 ± 0.01	−
	590–86-3	3-methylbutanal	−	−	2.56 ± 0.06
	111–71-7	heptanal	−	56.56 ± 0.29	2.25 ± 0.08
	98–01-1	furfural	−	−	3.68 ± 0.07
	620–02-0	5-methylfuran-2-carbaldehyde	−	−	1.91 ± 0.06
	106–26-3	(Z)-3,7-dimethylocta-2,6-dienal	−	−	0.94 ± 0.05
	5392–40-5	citral	−	0.07 ± 0.01	1.59 ± 0.07
	104–88-1	4-chlorobenzaldehyde	−	16.25 ± 0.53	0.86 ± 0.04
	25152–84-5	(E,E)-2,4-decadienal	−	38.80 ± 0.91	1.70 ± 0.03
	67–47-0	5-(hydroxymethyl) furan-2-carbaldehyde	−	31.11 ± 0.57	18.03 ± 0.09
	6728–26-3	(E)-2-hexenal	−	0.11 ± 0.01	−
	3913–81-3	(E)-2-decenal	−	68.89 ± 1.13	−
	20407–84-5	(E)-2-dodecenal	−	54.85 ± 0.62	−
		**grand total**	129.14 ± 1.26^b^	816.33 ± 9.96^a^	67.41 ± 0.64^c^
Alcohols	111–27-3	n-hexanol	41.06 ± 0.27	−	0.89 ± 0.01
	3391–86-4	1-octen-3-ol	21.87 ± 0.69^b^	58.48 ± 0.71^a^	3.36 ± 0.09^c^
	78–70-6	linalool	17.95 ± 0.73^b^	23.65 ± 0.71^a^	1.81 ± 0.06^c^
	18173–74-5	2-methoxyethanol	16.57 ± 0.85	−	−
	71–41-0	pentan-1-ol	−	5.49 ± 0.10	−
	98–55-5	α-terpineol	−	14.82 ± 0.59	1.27 ± 0.08
	5117–19-1	octaethylene glycol	−	0.26 ± 0.02	−
	546–79-2	2-methyl-5-(propan-2-yl) bicyclo[3.1.0]hexan-2-ol	−	5.68 ± 0.20	−
	98–00-0	furfuryl alcohol	−	9.43 ± 0.11	−
		**grand total**	97.45 ± 2.15^b^	117.82 ± 1.71^a^	7.32 ± 0.19^c^
Acids	79–31-2	2-methylpropanoic acid	−	−	2.84 ± 0.11
	107–92-6	butanoic acid	−	−	8.56 ± 0.42
	142–62-1	hexanoic acid	−	−	23.95 ± 2.70
	124–07-2	octanoic acid	−	−	23.17 ± 8.39
	334–48-5	decanoic acid	−	−	8.35 ± 0.14
	143–07-7	dodecanoic acid	−	−	18.01 ± 0.86
	503–74-2	3-methylbutanoic acid	−	−	33.35 ± 2.31
		**grand total**	−	−	118.23 ± 8.87
Ester	5259–87-0	methyl 2-(2-phenylacetamido) acetate	6.86 ± 0.12	−	−
	3055–98-9	octaethylene glycol monododecyl ester	78.31 ± 0.73^a^	27.28 ± 0.68^b^	3.57 ± 0.45^c^
	167871–30-9	tetradecyl 2-methylpropanoate	18.84 ± 0.96	−	−
	110–45-2	3-methylbutyl formate	−	−	5.49 ± 0.30
	89–48-5	(1R,2S,5R)-2-isopropyl-5-methylcyclohexyl acetate	−	0.75 ± 0.01	1.73 ± 0.07
	17851–53-5	butyl isobutyl phthalate	−	−	7.85 ± 0.11
	1962–75-0	dimethyl terephthalate	−	−	7.77 ± 0.13
	1867–95-4	octyl 2,2,3,3,3-pentafluoropropanoate	−	0.43 ± 0.07	−
	89–78-1	menthol	−	542.90 ± 50.77	11.74 ± 0.54
		**grand total**	104.01 ± 0.97^b^	571.35 ± 50.86^a^	38.16 ± 0.74^c^
Ketone	110–43-0	N-methylacetyl urea amyl ketone	1.22 ± 0.10	−	−
	110–12-3	5-methylhexan-2-one	4.88 ± 0.16	0.05 ± 0.01	−
	106–68-3	octan-3-one	15.73 ± 1.58	−	0.47 ± 0.01
	923–28-4	6-methylheptan-3-one	3.05 ± 0.11	−	−
	110–93-0	6-methylhept-5-en-2-one	8.53 ± 0.39^a^	4.04 ± 3.04^b^	2.05 ± 0.09^b^
	821–55-6	nonan-2-one	8.42 ± 0.26^a^	0.34 ± 0.19^c^	2.43 ± 0.26^b^
	18402–82-9	oct-3-en-2-one	5.72 ± 0.11	−	−
	1196–31-2	(+)-isomenthone	2.85 ± 0.08	−	2.27 ± 0.04
	112–12-9	undecan-2-one	11.59 ± 0.83	−	1.37 ± 0.05
	15932–80-6	5-methyl-2-(propan-2-ylidene) cyclohexan-1-one	−	61.74 ± 0.48	4.42 ± 0.11
	89–81-6	3-methyl-6-(propan-2-yl) cyclohex-2-en-1-one	−	−	4.95 ± 0.10
	2345–28-0	pentadecan-2-one	−	−	3.87 ± 0.10
	3214–41-3	2,5-octanedione	−	3.10 ± 0.03	−
	89–82-7	pulegone	−	0.44 ± 0.02	−
	624–42-0	6-methyl-3-heptone	−	−	0.92 ± 0.01
	491–07-6	isomenthone	−	360.16 ± 13.32	−
		**grand total**	61.77 ± 2.20^b^	419.86 ± 10.53^a^	22.73 ± 0.32^c^
Aromatic Compound	99–87-6	cymene	13.13 ± 0.85	−	−
	527–84-4	o-cymene	−	2.67 ± 0.09	1.86 ± 0.09
	535–77-3	p-cymene	−	0.15 ± 0.01	−
	95–47-6	1,2-dimethylbenzene	−	−	3.54 ± 0.09
	106–42-3	1,4-dimethylbenzene	−	−	0.48 ± 0.01
	108–88-3	toluene	−	−	0.12 ± 0.01
	1014–60-4	1,3-di-*tert*-butylbenzene	7.57 ± 0.75^b^	27.36 ± 2.05^a^	2.52 ± 0.15^c^
	59541–82-1	7-methyl-2-phenyl-1H-indole	−	−	1.44 ± 0.07
		**grand total**	20.70 ± 0.39^b^	30.17 ± 2.01^a^	9.97 ± 0.19^c^
Alkenes	5989–27-5	(+)-limonene	39.81 ± 4.30^a^	26.57 ± 4.95^b^	11.76 ± 5.27^c^
	469–61-4	(−)-α-cedrene	6.81 ± 0.41	5.97 ± 0.15	−
	475–20-7	longifolene	40.95 ± 9.85^a^	5.87 ± 0.17^b^	1.74 ± 0.10^b^
	17334–55-3	calarene	16.55 ± 1.81	−	−
	872–5-9	dec-1-ene	17.71 ± 0.88	−	−
	80–56-8	pinene	−	−	0.69 ± 0.01
	18172–67-3	(−)-β-pinene	−	−	4.92 ± 0.12
	2437–56-1	tridec-1-ene	−	−	1.99 ± 0.02
	821–95-4	1-hendecene	−	−	1.46 ± 0.11
	99–84-3	p-mentha-1(7),8-diene	−	4.47 ± 0.11	0.39 ± 0.01
	629–20-9	cyclooctatetraene	−	−	0.36 ± 0.04
	100–42-5	styrene	−	3.08 ± 0.10	0.49 ± 0.02
	694–87-1	benzocyclobutene	−	−	0.31 ± 0.02
	13360–61-7	pentadec-1-ene	−	−	2.75 ± 0.08
	87–44-5	β-caryophyllene	−	57.43 ± 20.08	2.09 ± 1.12
	13474–59-4	(E)-α-bergamotene	−	−	0.88 ± 0.03
	495–60-3	zingiberene	−	−	1.20 ± 0.15
	29050–33-7	4-carene	−	0.08 ± 0.11	−
	61142–36-7	3-ethyl-2-methylhexa-1,3-diene	−	16.67 ± 0.95	−
	644–30-4	α-curcumene	−	0.14 ± 0.01	−
	470–82-6	1,8-cineole	7.79 ± 0.84^b^	57.19 ± 7.47^a^	1.33 ± 0.09^b^
	4180–23-8	(E)-anethole	−	0.38 ± 0.04	−
		**grand total**	129.61 ± 15.68^a^	177.47 ± 24.99^a^	32.36 ± 6.55^b^
Phenols	108–39-4	m-cresol	−	−	8.35 ± 0.60
	106–44-5	p-cresol	−	−	3.95 ± 0.10
		2-pentylfuran	−	−	12.31 ± 0.53
Furan Compounds	3777–69-3	3-methylfuran	23.72 ± 1.79^a^	16.47 ± 1.26^b^	1.69 ± 0.16^c^
	930–27-8	menthofuran	−	−	0.86 ± 0.04
	494–90-6	dimethyl disulfide	−	16.81 ± 1.01	−
		grand total	23.72 ± 1.79^b^	33.29 ± 2.27^a^	2.55 ± 0.12^c^
Organic Sulfur	624–92-0	dimethyl trisulfide	22.47 ± 0.90	−	−
	3658–80-8	di-*tert*-dodecyl disulfide	15.65 ± 0.90	−	−
	27458–90-8	di-*tert*-dodecyl disulphide	7.73 ± 0.60	−	−
		**grand total**	45.85 ± 2.32	−	−
Other Kinds	91–20-3	naphthalene	17.09 ± 1.01	−	−
	3055–97-8	dodecyl heptaethylene glycol ether	0.98 ± 0.01	−	−
	19327–40-3	n-octyl pentoxy vinylacetylene	76.62 ± 3.54	0.28 ± 0.05	−
	112–50-5	2-(2-(2-ethoxyethoxy) ethoxy) ethanol	15.91 ± 0.65	47.50 ± 6.14	−
	23986–74-5	(−)-pyrethroid D	−	12.41 ± 1.44	−
	5695–49-8	1,3,5-trisilylhexane	−	0.44 ± 0.10	−
		**grand total**	110.61 ± 4.42	60.62 ± 4.95	−

Note: “-” indicates not detected.

Aldehydes, characterized by the presence of a carbonyl group, are formed through lipid oxidation, carbohydrate and amino acid degradation, and the oxidation of phenolic compounds [51]. As key flavor components in many foods, their generation and content in chicken are significantly influenced by cooking methods (e.g., boiling, frying, baking), thereby determining the final flavor profile. For instance, hexanal, nonanal, and 2,4-decadienal are key aldehydes in boiled chicken, imparting characteristic “meaty” and “fatty” notes [52]. Typical aldehydes in freshly cooked chicken, such as the long-chain compounds hexanal, (Z)-2-decenal, and (E)-2-decenal, have been identified as contributors to its core aroma profile [53]. Furthermore, nonanal and decanal have been confirmed as characteristic aroma substances in chicken [54]. The increase in aldehydes following UST is likely due to ultrasound's mechanical effects, which disrupt hydrogen bonds and electrostatic interactions within proteins. This leads to the unfolding and degradation of myofibrillar proteins, and the resulting peptides and free amino acids can subsequently undergo dehydration and condensation reactions to form aldehydes [55].

Esters, formed via esterification between carboxylic acids and alcohols, are key aroma compounds that impart fruity or floral notes in foods [56]. Their significance is evident in dishes such as salt-baked chicken, where aldehydes and esters dominate the flavor profile [57], and in contributing to the balanced taste of ginkgo chicken soup [58]. Notably, during stewing, esters—along with alcohols and heterocyclic flavors—mainly originate from interactions between spice compounds and chicken tissues, unlike aldehydes and ketones derived from lipid oxidation [59]. The increase in esters following UST can be attributed to two mechanisms: firstly, ultrasound disperses insoluble reactants (e.g., fatty acids and alcohols) to form homogeneous emulsions, increasing interfacial contact and accelerating esterification [60]; secondly, cavitation and mechanical vibration enhance molecular collisions, promoting early Maillard reaction steps that generate aldehyde intermediates for subsequent ester formation [61].

Ketones, characterized by a carbonyl group bonded to two carbon atoms, significantly influence flavor by interacting with other compounds and food matrices (e.g., proteins, lipids), thereby modulating flavor release and perception [62]. They are generated through pathways including amino acid degradation, lipid oxidation, carbohydrate metabolism, and beta-keto acid oxidation [63]. In roasted chicken, ketones contribute creamy and fruity notes [64], and they, along with aldehydes and alcohols, have been identified as dominant flavor contributors in both breast and leg muscles of Lueyang black-bone chickens [65]. The increase in ketones following UST is attributed to two main mechanisms. First, ultrasound facilitates the formation of precursors (e.g., from amino acid degradation) that convert to ketones during cooking [10]. Second, the mechanical and thermal effects of ultrasound induce protein denaturation and degradation, exposing more amino acids and peptides. These Maillard reaction precursors are more likely to react with reducing sugars during heating, thereby promoting ketone formation [66].

Acidic compounds in food science are sour-tasting substances capable of donating protons and reacting with bases. They serve critical flavor functions, including imparting sourness, regulating pH and flavor balance, promoting aroma release, and enhancing saltiness perception [67]. These acids originate from natural biosynthesis, microbial fermentation, industrial extraction, or synthetic pathways [68], [69], [70]. In chicken soup, succinic and lactic acids significantly contribute to the overall taste profile by interacting with other flavor compounds [1]. The increase in acidic compounds following HPH is likely due to the substantial reduction in fat globule size, which increases the oil–water interfacial area. This accelerated interfacial contact promotes the oxidation of unsaturated fatty acids, leading to the formation of carboxylic acids as oxidation products [71].

Phenolic compounds are defined as organic molecules containing at least one hydroxyl group attached to an aromatic ring. They contribute diverse flavor characteristics, including astringency (tannins), bitterness (naringin), and spicy aromas [72]. Beyond their direct sensory impact, they modulate flavor through multiple mechanisms: exerting antioxidant activity to preserve original flavors, participating in enzymatic interactions (e.g., polyphenol oxidase-mediated browning), and binding with proteins, carbohydrates, and lipids [73], [74], [75]. The increase in phenolic compounds following HPH may be attributed to intense shear forces, cavitation, and impact effects that disrupt cellular structures, thereby releasing encapsulated phenolics into the soup [76]. Additionally, HPH may induce structural changes in macromolecules (e.g., proteins, carbohydrates), exposing new binding sites for phenolics or facilitating the conversion of bound precursors into free forms via enzymatic or hydrolytic pathways [77]. In summary, UST significantly increased the concentrations of aldehydes, esters, and ketones in the chicken soup, whereas HPH markedly elevated the levels of acids and phenolic compounds. These findings demonstrate that both techniques are effective methods for enhancing the content of key flavor compounds, thereby improving the soup's sensory profile and leading to a superior overall flavor.

#### 3.4.2 Analysis of odor activity value

The contribution of VFCs was quantitatively assessed by calculating their OAVs, with the full results detailed in Table S4. Based on their OAVs, compounds were categorized as key flavor components (OAV > 1), modifying components (0.1 ≤ OAV ≤ 1), or components with negligible contributions (OAV < 0.1). The results indicate that the U group significantly enriched the soup's profile by increasing the number of key aldehydes, such as (E)-2-decenal and (E)-2-dodecenal, compared to the C group. In contrast, the H group not only retained several key aldehydes but also introduced isovaleraldehyde as a key component and exhibited a greater number of modifying components. For a more intuitive comparison, the normalized OAV data are visualized in a bubble plot (Fig. S1), which clearly illustrates considerable differences among the groups, suggesting distinct overall flavor profiles resulting from the different processing techniques. Analysis of the OAV data (Table S4, Fig. S1) identified the six most potent odorants in each group. In the U group, these were (E,E)-2,4-decadienal (OAV = 494.55), (E)-2-decenal (229.63), (E,E)-2,4-nonadienal (211.10), linalool (107.50), hexanal (57.42), and 1,8-cineole (51.99), which collectively contributed attributes described as oily, roasted, sweet, fatty-oxidized, floral, and sweet aromatic. The corresponding compounds in the H group were (E,E)-2,4-decadienal (22.08), toluene (10.91), linalool (8.22), nonanal (3.72), decanal (3.56), and 3-methylbutanal (2.33), providing sweet, fatty-oxidized, toasted, and oily characteristics. In the C group, the highest OAVs were for dimethyl trisulfide (156.50), (E,Z)-2,4-decadienal (149.50), linalool (81.59), (E,E)-2,4-nonadienal (25.1), dimethyl disulfide (20.43), and 1-octen-3-ol (14.58), associated with savory, floral, sweet, umami, mushroom, and meaty flavors. Critically, the absolute OAV values were significantly greater in the U group than in the C and H groups, indicating a more pronounced flavor impact. This molecular-level evidence directly correlates with the sensory analysis results, thereby offering a mechanistic explanation for the superior sensory scores awarded to the U group.

#### 3.4.3 Analysis of multivariate statistical

**Fig. 5:**
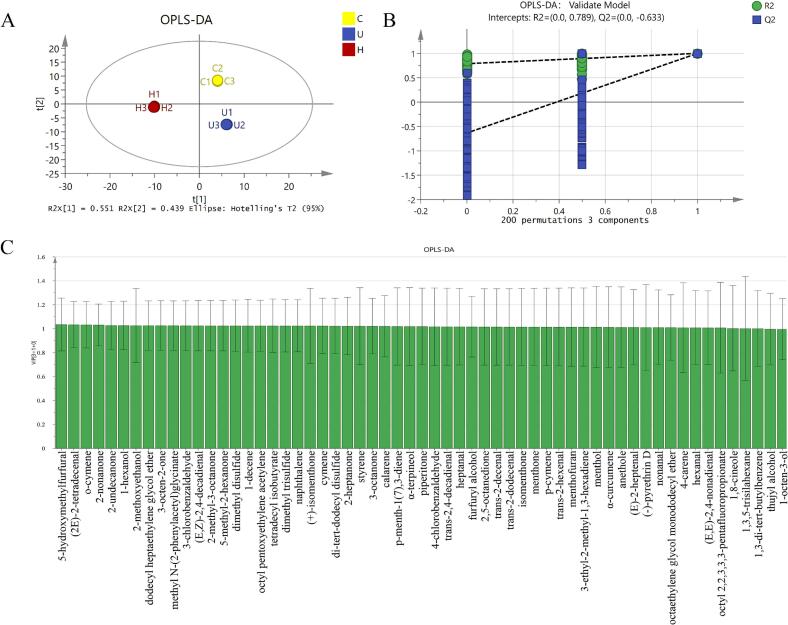
Multivariate statistical analysis based on GC–MS. (A) score plot, (B) 200 permutation test plot, (C) VIP > 1 compounds plot. In Figure (A), the x- and y-axes both represent the on-chart distance, with different colored blocks corresponding to different experimental groups. An annotation at the bottom provides the model’s equation and its explained variance (95%). Figure (B) also uses on-chart distance for both axes, where the green and blue elements depict the R2 and Q2 values, respectively, for different flavor compounds; the text at the top lists the model’s parameters. Figure (C) plots the variable importance for the projection (VIP) scores on the y-axis against the corresponding compound names on the x-axis.

The OPLS–DA model, a supervised pattern-recognition method designed to maximize separation between predefined sample groups [78], was employed to analyze overall flavor differences and identify key discriminant compounds in the chicken soup samples, with variable selection based on VIP scores (Fig. 5). As shown in the score plot (Fig. 5A), samples within each group (C, U, H) clustered closely, indicating minimal intra-group variation and high experimental reproducibility. Concurrently, the distinct separation of the three groups in the score plot demonstrated significant inter-group differences in their flavor compound profiles. Notably, both the U and H groups were positioned distantly from the C group, confirming that both treatments substantially altered the soup's flavor characteristics. The model's goodness-of-fit was assessed using R-squared (R2), which measures the proportion of variance explained by the model, with values closer to 1 indicating a better fit [79]. To validate the model's reliability and prevent overfitting, a 200-permutation cross-validation test was performed. The validation results (Fig. 5B) yielded an R^2^ value of 0.789, with the permutation test plot showing all permuted R^2^ and Q^2^ values to the left lower than the original point to the right, and the regression line of Q^2^ intersecting the vertical axis below zero. These indicators confirm that the model is robust and reliable without overfitting [80].

VIP scores from the OPLS-DA model were used to identify compounds significant for discriminating the sample groups, with a VIP > 1.0 indicating major contributors. A total of 57 compounds met this criterion (Table S5, Fig. 5C). Cross-referencing with the compositional data revealed that five compounds—5-hydroxymethylfurfural, o-cymene, styrene, p-menth-1(7),3-diene, and α-terpineol—were exclusively detected in the U and H groups, suggesting their role as potential markers distinguishing these treatments from the conventional method. 5-hydroxymethylfurfural, a furan derivative, is primarily formed via the Maillard reaction or sugar caramelization under thermal/acidic conditions, imparting caramel-like aromas [81], and is noted for various bioactivities [82]. Its formation during ultrasonic and HPH treatments can be attributed to the creation of localized high-temperature microenvironments: ultrasonic cavitation generates extreme heat and pressure upon bubble collapse [83], while HPH induces frictional heating under high pressure [84]. Both mechanisms facilitate the reactions leading to 5-hydroxymethylfurfural generation. In summary, OPLS-DA analysis revealed distinct overall flavor characteristics among the C, U, and H groups, with compounds including 5-hydroxymethylfurfural, o-cymene, styrene, p-menth-1(7),3-diene, and α-terpineol identified as key discriminatory markers distinguishing the U and H groups from C group.

### 3.5 Correlation analysis between flavor precursors and OAV flavor compounds

**Fig. 6:**
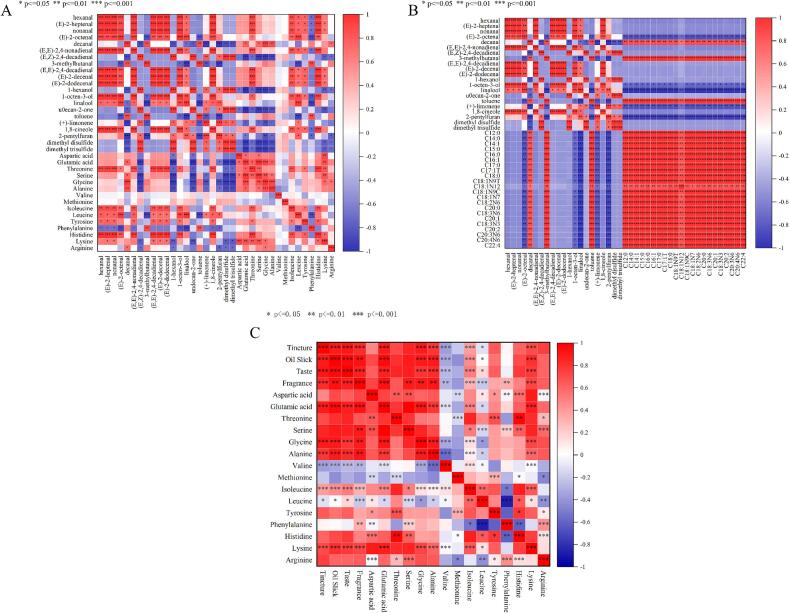
Correlation analysis between flavor precursors and key volatile flavor compounds. (A) Correlations between free amino acids and volatile flavor compounds; (B) Correlations between free fatty acids and volatile flavor compounds. (C) Correlations between free amino acids and sensory evaluation. “*” *p* < 0.05, “**” *p* < 0.01, “***” *p* < 0.001. In Figures (A) through (C), both the x- and y-axes represent the compounds. The heatmap is color-coded to indicate the strength of correlation between them, with a gradient from blue (low correlation) to red (high correlation).

Amino acids undergo catabolic degradation under high pressure and heating conditions, producing small-molecule peptides and free amino acids, which serve as important precursors for key flavor components, including aldehydes, alcohols, ketones, and sulfur-containing compounds [85]. Fatty acids (FAs) are another major precursor of volatile flavor compounds (VFCs), as their thermally induced oxidation—particularly of unsaturated fatty acids—generates degradation products such as aliphatic aldehydes, ketones, and alcohols [86]. Pearson correlation analysis was conducted to examine the relationships between 21 odor activity value (OAV) flavor compounds and flavor precursors, including fatty acids and amino acids. The results of the correlation analysis between free amino acids and key volatile flavor compounds (Fig. 6A) revealed significant positive correlations (P < 0.05) between amino acids such as threonine, isoleucine, leucine, tyrosine, histidine, and lysine and key volatile flavor compounds including 1,8-cineole, 1-octen-3-ol, (E,E)-2,4-decadienal, (E)-2-decenal, (E)-2-dodecenal, (E,E)-2,4-nonadienal, hexanal, (E)-2-heptenal, nonanal, and (E)-2-octenal. Significant negative correlations were observed between amino acids such as glutamic acid, threonine, serine, glycine, alanine, lysine, and arginine and compounds including (E,Z)-2,4-decadienal, 1-hexanol, undecan-2-one, (+)-limonene, dimethyl disulfide, and dimethyl trisulfide. Aldehydes can be formed through the Maillard reaction between the amino group of free amino acids and the carbonyl group of sugars [86], while α-amino acids and carbonyl compounds can also undergo Strecker degradation, producing a variety of flavor-characteristic aldehydes [87]. Heat treatment promotes the Maillard reaction [88]. In the present study, most of the compounds showing positive correlations with amino acids were aldehydes, which may be attributed to the Maillard reaction between the aforementioned amino acids and sugars under high-temperature stewing conditions, leading to the formation of these aldehydes.

A correlation analysis was performed between free fatty acids and key volatile flavor compounds that were present in all three groups and had concentrations exceeding 1 μg/mL in at least one group. The results are shown in Fig. 6B. Compounds including decanal, 3-methylbutanal, and toluene exhibited highly significant positive correlations (P < 0.01) with all selected fatty acids, while (E)-2-octenal, 1-octen-3-ol, linalool, (+)-limonene, and 2-pentylfuran showed significant negative correlations (P < 0.05) with the fatty acids. Decanal is a secondary product of lipid oxidation or thermal degradation, formed through the oxidation of unsaturated fatty acids such as oleic, linoleic, or linolenic acid via hydroperoxide intermediates [89], [90]. The oxidative decomposition of fatty acids yields various volatile compounds, including unsaturated aldehydes such as (E)-2-octenal. The negative correlation observed between these aldehydes and fatty acid content suggests that their formation decreases as the concentration of fatty acids increases [91]. In summary, both amino acids and fatty acids serve as important flavor precursors in chicken broth and exert a significant influence on the formation of key volatile flavor compounds.

Meanwhile, sensory evaluation results indicated that the U group received the highest scores in taste perception. Concurrently, amino acid analysis also showed increases in umami and sweet taste-related amino acids. Therefore, a correlation analysis between amino acids and sensory evaluation was performed, with the results presented in Fig. 6C. Sensory attributes, including tincture, oil slick, taste, and fragrance, showed positive correlations with aspartic acid, glutamic acid, threonine, serine, glycine, alanine, histidine, lysine, and arginine. Among these, extremely significant correlations (*p* < 0.01) were observed with glutamic acid, glycine, alanine, and lysine. Furthermore, the aforementioned four sensory attributes exhibited negative correlations with valine and methionine, with the correlation with valine being extremely significant (*p* < 0.01). Furthermore, combined with the analysis of amino acid content and TAV in section 3.3 above, histidine was defined as a key amino acid because it showed the highest content and had a TAV > 1 in all three groups. Meanwhile, glutamic acid content was also found to be higher in the U and H groups than in the C group, and the same trend was observed for its TAV value. It has also been reported that glutamic substances can stimulate saliva secretion, increase oral lubrication, and sustain the persistence of umami in the oral cavity [92]. Min et al. [93] found that an increase in glutamic acid content directly and significantly enhanced the umami intensity of Hanwoo beef, which is consistent with the findings of this study, namely that an increase in glutamic acid content leads to an improvement in taste (umami). The study by Bi et al. [94] found that glutamic acid content decreased significantly with prolonged refrigerated storage (dropping to about 50% of the initial level by day 10), a change that directly led to a reduction in the umami intensity of cooked Tan sheep meat and a more pronounced bitterness, thereby overall reducing the flavor quality of the meat product. Studies have also shown that the taste expression of amino acids is complex, often presenting mixed taste sensations, such as certain amino acids simultaneously eliciting sweet and bitter tastes, depending on their concentration, solution environment, and individual genetic variations [95]. Regarding histidine specifically, in this study, due to its higher concentration, it may have a significant impact on the taste attributes (including umami, sweetness, etc.) in the sensory experience. Furthermore, correlation analysis revealed that both glutamic acid and histidine showed positive correlations with the taste evaluation scores in the sensory assessment. This suggests that the superior taste performance of U and H groups, compared to C group, is potentially due to their higher concentrations of glutamic acid and histidine. The positive correlation of these amino acids with sensory taste scores, along with their likely synergistic effects with other amino acids, may explain the observed difference in overall taste perception.

Furthermore, although the present study did not involve research on endogenous enzyme activity, the formation of flavor in meat products is a complex process in which endogenous enzymes play a crucial role [96]. Endogenous enzymes in food, such as lipases, lipoxygenases, proteases, and glycosidases, degrade macromolecules (e.g., fats, proteins, carbohydrates) during production, processing, storage, and even digestion, thereby generating flavor precursors or direct flavor compounds [97]. Meanwhile, endogenous enzymes influence the release of important flavor precursors, such as FAAs and FFAs. Lipolysis is defined as the process in which triglycerides are hydrolyzed by lipases, releasing FFAs and glycerol [98]. Lipases, as key components of endogenous enzymes, catalyze the hydrolysis of triglycerides into FFAs. This process involves substrate recognition and binding, formation of an acyl-enzyme intermediate, hydrolysis of the intermediate, and finally, the release of FFAs along with regeneration of the free lipase, enabling it to catalyze subsequent reactions [99]. FAAs are mainly derived from protein degradation. Proteolysis refers to the cleavage of peptide bonds in proteins by proteases, releasing FAAs, peptides, and polypeptides [100]. The conversion of proteins into FAAs primarily involves transamination and deamination. Transamination is a key process in amino acid metabolism, in which transferases (also known as aminotransferases) transfer an amino group from an amino acid to an α-keto acid, forming a new amino acid and α-keto acid. Deamination refers to the removal of an α-amino group from an amino acid, yielding the corresponding α-keto acid and free ammonia [101].

Li et al. [102] investigated the potential relationship between endogenous proteases and key flavor compounds in dry-cured pork rolls (DDPs). The results indicated that the activities of endogenous proteases (such as calpain, cathepsins, dipeptidyl peptidases, and aminopeptidases) showed dynamic changes during processing, with cathepsins and DPPs playing a dominant and continuous role in proteolysis. These enzymes significantly influenced flavor formation by regulating the accumulation of FAAs (e.g., Glu, Leu, Val) and volatile compounds (e.g., hexanal, ethyl octanoate). Guo et al. [103] studied the dynamics of endogenous enzyme activities (including lipase, lipoxygenase, and antioxidant enzymes), lipid oxidation indicators, and volatile flavor compounds during the processing of Xinjiang dry-cured mutton ham. The results demonstrated that lipase and lipoxygenase directly promoted the formation of key flavor compounds such as aldehydes (e.g., hexanal) and alcohols by catalyzing lipid hydrolysis and oxidation, while antioxidant enzymes (e.g., superoxide dismutase) maintained flavor balance by regulating the degree of oxidation. In summary, endogenous enzymes have a significant influence on the flavor of meat products. In the future, if UST and HPH are to be applied on a large scale in chicken soup production, further investigation into the effect of endogenous enzyme activity on the quality of chicken soup will be necessary to ensure consistency in the final product quality.

### 3.6 A comprehensive analysis of the effects of ultrasonic treatment and high-pressure homogenization on the quality of chicken soup

As described above, UST significantly improved sensory scores and the content of key flavor compounds, while HPH increased the content of FFAs. To explain these observations, a thorough review of the literature was conducted. It was found that this phenomenon may be attributed to the influence of ultrasonic cavitation and high-pressure shear forces on protein denaturation, lipid oxidation, and the Maillard reaction in chicken soup, with potential synergistic or competitive effects between the two mechanisms. When ultrasonic waves propagate through a liquid, micro-bubbles are generated, which undergo growth, oscillation, and rapid collapse under acoustic pressure—a process known as cavitation [104]. The collapse of these bubbles produces localized extreme temperatures (up to thousands of Kelvin) and pressures (up to hundreds of megapascals), accompanied by high-speed micro-jets and shear forces. In this study, HPH was applied as an independent mechanical treatment in food processing, which alters material structure through mechanical action. Meanwhile, high-pressure shear forces are also typically an accompanying effect of the ultrasonic cavitation process [105]. The high temperature, high pressure, and mechanical shear forces generated by ultrasonic cavitation can induce conformational changes in proteins, leading to protein denaturation. For example, Hong et al. [106] found that high-intensity ultrasound (HIU) reduced the size of fish myofibrillar proteins (MP), unfolded their conformations, and exposed more hydrophobic groups, thereby promoting protein denaturation. Quaisie et al. [105] reported that the high-pressure shear forces generated by dual-frequency ultrasound (SDFU) could cause disentanglement, unfolding, and aggregation of protein molecular chains through mechanical action, resulting in denaturation. Furthermore, studies have indicated that the high temperature and pressure from ultrasonic cavitation can accelerate the unfolding of protein molecules, while the accompanying high-pressure shear forces can further disaggregate protein aggregates, collectively promoting protein denaturation and thereby improving functional properties such as solubility and emulsification stability [107]. In summary, since both UST and HPH generate shear forces, they may exhibit a synergistic effect in terms of protein denaturation.

In terms of lipid oxidation, the free radicals (such as hydroxyl radicals) generated during ultrasonic cavitation may accelerate lipid oxidation. Wang et al. [108] found that UST significantly promoted fat release and oxidation in salted egg yolk through cavitation and thermal effects. This is likely because the high-temperature, high-pressure environment and free radicals produced by ultrasonic cavitation promoted the oxidation of unsaturated fatty acids, thereby generating VFCs such as aldehydes and ketones. High-pressure treatment can induce the oxidation of lipids and proteins, promoting the formation of free radicals [109]. Under high-pressure shear forces, fat globule membranes may rupture, increasing the contact area between lipids and oxidants and accelerating the oxidation process. Auñon-Lopez et al. [110] found that during ultra-high-pressure homogenization of emulsions, the disruption of the interfacial membrane may lead to an increase in FFA content and the accumulation of lipid oxidation products. In this study, the H group showed a higher FFA content than the U group. This may be because the mechanical forces generated by HPH effectively break large fat globules into smaller particles, significantly increasing the surface area of the fat. Meanwhile, it may not completely inactivate lipases; instead, it may alter enzyme conformation or expose active sites, making it easier for lipases to bind to lipid substrates, thereby promoting their hydrolytic activity [110] and further enhancing FFA generation. In contrast, the localized high temperature and free radicals produced by UST may cause irreversible denaturation or inactivation of enzymes [111], resulting in a lower FFA content compared to the H group. Furthermore, the discrepancy between the findings of Yu et al. [111] and those of Wang et al. [108] mentioned above may be attributed to differences in UST conditions and the products processed. In summary, UST and HPH may exhibit competitive effects in terms of lipid oxidation and FFA generation, with HPH demonstrating superior effectiveness compared to UST.

Regarding the Maillard reaction, the high-temperature and high-pressure environment generated by UST is favorable for the reaction between sugars and amino compounds. Furthermore, UST can accelerate the Maillard reaction by altering protein structures, such as increasing surface hydrophobicity and exposing reactive sites [112]. Yu et al. [113] demonstrated that in a model system of D-glucose and glycine under high-intensity ultrasound-assisted Maillard reaction, the formation of dicarbonyl compounds was significantly promoted, leading to an increased generation rate of intermediate and final Maillard reaction products. Wang et al. [108] also found that UST indirectly enhanced the Maillard reaction process by accelerating water evaporation, protein denaturation, and lipid oxidation. Their GC‒MS data further indicated that the contents of aldehydes (e.g., benzaldehyde, octadecanal) and phenolic substances were elevated in ultrasonic-treated samples, with some of these compounds derived from interactions between Maillard reaction intermediates and lipid oxidation products. HPH primarily relies on strong shear and impact forces to refine particles and emulsify systems. These mechanical forces can trigger a series of physical and chemical changes, thereby affecting the Maillard reaction [114]. Li et al. [114] reported that in black garlic processing, high-pressure pretreatment disrupted cellular structures, promoted enzymatic reactions, and consequently accelerated the Maillard reaction, improving product quality. The Maillard reaction is a major source of many important VFCs in foods, including pyrazines, aldehydes, and ketones, which impart distinctive aromas, such as those characteristic of roasted meat, baked goods, and coffee [115]. Heat treatment serves as the core condition for initiating and accelerating the Maillard reaction, profoundly regulating flavor formation in foods by influencing reaction rate, product distribution, and reaction pathways [115]. UST can rapidly initiate and accelerate the Maillard reaction in a short time through cavitation and thermal effects, especially under low-temperature conditions, where localized high-energy zones and radical-induced effects are more pronounced [113]. This explains why UST outperformed HPH in increasing flavor compounds in this study. In summary, in terms of the Maillard reaction, a potential competitive effect may exist between UST and HPH.

## 4 Conclusion

This study investigated the effects of different processing methods on the quality and flavor characteristics of ​​Wuliang Mountain black-boned chicken​ soup. Three treatments were applied: conventional atmospheric stewing (C group), UST (U group), and HPH (H group). Key indicators including sensory scores, FAAs, FFAs, and VFCs were analyzed using an amino acid analyzer, GC–MS. Results demonstrated that the U group achieved the highest overall and individual sensory scores. UST significantly enhanced key flavor-active amino acids such as glutamic acid, lysine, and histidine, resulting in a marked increase in total FAAs (*p* < 0.05), while the H group showed more pronounced elevations in phenylalanine and glycine. The fatty acid content in the H group was significantly higher than in the other groups (*p* < 0.05), with palmitic acid, oleic acid, and linoleic acid levels reaching 21.6, 41.8, and 47.5 times those of the C group, respectively. A total of 107 VFCs were identified across all samples. The U group exhibited significantly higher concentrations of aldehydes, esters, and ketones, whereas the H group showed elevated levels of acids and phenols. Moreover, the U group had significantly higher OAV than the C and H groups, suggesting superior flavor expression. VIP analysis identified 57 compounds with VIP > 1, among which 5-hydroxymethylfurfural, o-cymene, styrene, p-menth-1(7),3-diene, and α-terpineol were unique to the U and H groups, serving as key discriminative flavor markers distinguishing them from the C group. Pearson correlation analysis indicated that amino acids and fatty acids in the chicken soup had significant effects on the volatile flavor compounds. In conclusion, UST significantly improved sensory quality and flavor compound content, while HPH markedly increased free fatty acid levels. These findings provide a scientific basis for resource development, utilization, and conservation of Wuliang Mountain black-boned chicken.

## Data availability

Data will be made available on request.

## CRediT authorship contribution statement

**Shuaihan Jiang:** Writing – original draft, Investigation, Conceptualization. **Hongqin Chen:** Writing – review & editing, Writing – original draft, Software, Data curation. **Sisi Zhang:** Software, Data curation. **Zhiqiang Xu:** Writing – review & editing, Supervision, Funding acquisition, Conceptualization. **Shijun Li:** Supervision, Project administration.

## Declaration of competing interest

The authors declare that they have no known competing financial interests or personal relationships that could have appeared to influence the work reported in this paper.
