# Supplementary material for: Evaluating the effects of ultrasonic and high-pressure homogenization on the flavor profile of Wuliang Mountain black-boned chicken soup
Source: Ultrason Sonochem. 2026 Mar 4;128:107810. doi: 10.1016/j.ultsonch.2026.107810 (PMC12992082; doi:10.1016/j.ultsonch.2026.107810)
Supplement: Supplementary Data 1 [file mmc1.docx]

Supplementary material

Table S1 The sensory rating table

| Scoring criteria | Standard for evaluation | Score |
| --- | --- | --- |
| Color | Milky white or light yellow | 17~25 |
|  | Beige and light brownish yellow | 9~16 |
|  | Grayish white or colorless | 0~8 |
| Oil slick | No obvious floating oil | 17~25 |
|  | Little surface oil | 9~16 |
|  | Excessive surface oil | 0~8 |
| Flavor | Rich taste, lingering aftertaste, appropriate umami | 17~25 |
|  | Insufficient umami, pure and authentic taste | 9~16 |
|  | Light flavor, no aftertaste, poor umami | 0~8 |
| Fragrance | Strong meaty aroma | 17~25 |
|  | Slightly weak meaty aroma | 9~16 |
|  | Relatively weak meaty aroma | 0~8 |

Table S2 Amino acid composition and content

| Flavor | Name | Amino Acid Composition and Content（mg/L） | | |
| --- | --- | --- | --- | --- |
|  |  | C group | U group | H group |
| ​Umami​ | Aspartic acid（Asp） | 7.23±0.29^a^ | 7.63±0.06^a^ | 7.50±0.20^a^ |
|  | Glutamic acid（Glu） | 22.50±0.69^b^ | 32.03±1.50^a^ | 31.03±0.23^a^ |
|  | Lysine（Lys） | 10.40±0.35^b^ | 11.77±0.46^a^ | 11.20±0.36^a^ |
|  | ​​Subtotal | 40.13±1.33^b^ | 51.43±1.99^a^ | 49.73±0.57^a^ |
| ​​Sweetness​ | Threonine（Thr） | 8.97±0.40^c^ | 19.07±0.72^a^ | 12.07±0.93^b^ |
|  | Serine（Ser） | 10.87±0.40^b^ | 15.03±0.93^a^ | 14.57±0.67^a^ |
|  | Glycine（Gly） | 7.60±0.17^b^ | 9.10±0.10^a^ | 9.53±0.42^a^ |
|  | Alanine（Ala） | 12.63±0.12^b^ | 14.57±0.87^a^ | 14.90±0.66^a^ |
|  | Histidine（His） | 233.40±1.99^c^ | 314.23±3.58^a^ | 240.90±1.90^b^ |
|  | ​Subtotal | 273.47±1.76^c^ | 372.00±3.84^a^ | 291.97±1.21^b^ |
| Bitterness​ | Valine（Val） | 19.93±0.29^a^ | 19.63±1.02^a^ | 19.53±0.90^a^ |
|  | Methionine（Met） | 3.57±0.58^a^ | 3.43±0.15^a^ | 3.30±0.44^a^ |
|  | Isoleucine（Ile） | 3.17±0.23^b^ | 4.10±0.30^a^ | 3.07±0.38^b^ |
|  | Leucine（Leu） | 7.23±0.12^b^ | 7.93±0.50^a^ | 5.73±0.12^c^ |
|  | Arginine（Arg） | 5.57±0.46^b^ | 6.07±0.25^ab^ | 6.43±0.31^a^ |
|  | ​Subtotal | 39.47±0.23^b^ | 41.17±0.80^a^ | 38.07±1.07^b^ |
| ​​Aromatic | Tyrosine（Tyr） | 4.90±0.26^b^ | 5.57±0.58^a^ | 4.97±0.45^ab^ |
|  | Phenylalanine（Phe） | 5.50±0.46^b^ | 4.97±0.25^b^ | 7.00±0.56^a^ |
|  | ​Subtotal | 10.40±0.26^b^ | 10.53±0.21^b^ | 11.97±0.47^a^ |
|  | ​​Total Free Amino Acids | 363.47±2.42^c^ | 475.13±6.05^a^ | 391.73±1.59^b^ |

Note: The lowercase letters ' a, b, c ' indicate that there is a significant difference between peers, P < 0.05.

Table S3 Amino acid threshold and TAV

| Flavor | Name | Threshold value（mg/L） | TAV of amino acids in chicken soup | | |
| --- | --- | --- | --- | --- | --- |
|  |  |  | C group | U group | H group |
| Umami | Aspartic acid（Asp） | 1000 | 0.007 | 0.008 | 0.008 |
|  | Glutamic acid（Glu） | 300 | 0.075 | 0.107 | 0.103 |
|  | Lysine（Lys） | 500 | 0.021 | 0.024 | 0.022 |
| Sweetness | Threonine（Thr） | 2600 | 0.003 | 0.007 | 0.005 |
|  | Serine（Ser） | 1500 | 0.007 | 0.010 | 0.010 |
|  | Glycine（Gly） | 1300 | 0.006 | 0.007 | 0.007 |
|  | Alanine（Ala） | 600 | 0.021 | 0.024 | 0.025 |
|  | Histidine（His） | 200 | 1.167 | 1.571 | 1.205 |
| Bitterness | Valine（Val） | 400 | 0.050 | 0.049 | 0.049 |
|  | Methionine（Met） | 300 | 0.012 | 0.011 | 0.011 |
|  | Isoleucine（Ile） | 900 | 0.004 | 0.005 | 0.003 |
|  | Leucine（Leu） | 1900 | 0.004 | 0.004 | 0.003 |
|  | Arginine（Arg） | 500 | 0.011 | 0.012 | 0.013 |
| Aromatic | Tyrosine（Tyr） | 500 | 0.010 | 0.011 | 0.010 |
|  | Phenylalanine（Phe） | 900 | 0.006 | 0.006 | 0.008 |

Table S4 odor characteristics of flavor substances and OAV table

| Name of Compound | Smell Characteristics | Aroma Description | threshold value（μg/L） | OAV | | |
| --- | --- | --- | --- | --- | --- | --- |
|  |  |  |  | C group | U group | H group |
| hexanal | Grass, fat flavor, green apple flavor | Oily, baking, light salty, a little floral and fruity. | 5 | 7.48 | 57.42 | 1.24 |
| (E)-2-heptenal | Oxidized fat, grass, nutty aroma | Oily, roasted, caramel-scented salty aroma | 40 | 0.22 | 1.27 | 0.16 |
| nonanal | Orange, fat, wax aroma | Salty, light chocolate and oily aromas of baked coffee | 1.1 | 11.54 | 101.83 | 3.72 |
| (E)-2-octenal | Fat, grass, mushroom flavor | Bitter almond flavor, nut flavor accompanied by meat flavor | 3 | 7.97 | 16.35 | ND |
| decanal | Orange peel, soap, fat flavor | Baking flavor, creamy fat aroma | 3 | 2.64 | 3.10 | 3.56 |
| (E,E)-2,4-nonadienal | Fried flavor, fat oxidation flavor, cucumber peel | Salty butter aroma | 0.1 | 25.1 | 211.10 | ND |
| (E,Z)-2,4-decadienal | Fried potatoes, fat, metal flavor | Saline fragrance | 0.04 | 149.50 | 2.75 | ND |
| 3-methylbutanal | Malt flavor, fruit flavor, fermentation flavor | Baking bread | 1.1 | ND | ND | 2.33 |
| (E,E)-2,4-decadienal | Fried flavor, fat oxidation flavor | Sweet, fat oxidation flavor | 0.077 | ND | 494.55 | 22.08 |
| (E)-2-decenal | Fat, wax, citrus | - | 0.3 | ND | 229.63 | ND |
| (E)-2-dodecenal | Metallic flavor, fat oxidation flavor | - | 1.4 | ND | 39.18 | ND |
| 1-hexanol | Grass, herbs, slight oil flavor | The aroma of wheat | 5.6 | 7.33 | ND | 0.16 |
| 1-octen-3-ol | Mushroom, soil, metal flavor | Saline, mushroom flavor, meat flavor | 1.5 | 14.58 | 38.99 | 2.24 |
| linalool | Flower fragrance, citrus, lavender | Flowery, sweet fragrance | 0.22 | 81.59 | 107.50 | 8.22 |
| undecan-2-one | Waxy, fruity (similar to blueberry) | - | 5.5 | 2.11 | ND | 0.25 |
| toluene | Solvent flavor, sweetness | Toasty bread flavor | 0.011 | ND | ND | 10.91 |
| (+)-limonene | Orange peel, pine wood flavor | Baking flavor | 34 | 1.17 | 0.78 | 0.35 |
| 1,8-cineole | Mint, camphor, cool feeling | Sweet aroma | 1.1 | 7.08 | 51.99 | 1.21 |
| 2-pentylfuran | Beany flavor, grass, slight fruity. | Aroma and milky aroma of pepper salty sweet spices | 5.8 | 4.09 | 2.84 | 0.29 |
| dimethyl disulfide | Sulfur, cabbage flavor | - | 1.1 | 20.43 | ND | ND |
| dimethyl trisulfide | Sulfur, garlic | fishy smell | 0.1 | 156.50 | ND | ND |

Table S5 Differential flavor substances in three kinds of chicken soup

| Number | Compounds name | VIP | P. value |
| --- | --- | --- | --- |
| 1 | 5-hydroxymethylfurfural | 1.03496 | 0.221037 |
| 2 | (2E)-2-tetradecenal | 1.03387 | 0.193248 |
| 3 | o-cymene | 1.03337 | 0.1942 |
| 4 | 2-nonanone | 1.0321 | 0.173384 |
| 5 | 2-undecanone | 1.02698 | 0.200469 |
| 6 | 1-hexanol | 1.02693 | 0.202824 |
| 7 | 2-methoxyethanol | 1.02624 | 0.310156 |
| 8 | dodecyl heptaethylene glycol ether | 1.02617 | 0.206545 |
| 9 | 3-octen-2-one | 1.02605 | 0.207701 |
| 10 | methyl N-(2-phenylacetyl) glycinate | 1.026 | 0.206548 |
| 11 | 3-chlorobenzaldehyde | 1.02583 | 0.207406 |
| 12 | (E,Z)-2,4-decadienal | 1.02551 | 0.210785 |
| 13 | 2-methyl-3-octanone | 1.02539 | 0.21054 |
| 14 | 5-methyl-2-hexanone | 1.02524 | 0.210884 |
| 15 | dimethyl disulfide | 1.02522 | 0.214677 |
| 16 | 1-decene | 1.02508 | 0.220553 |
| 17 | octyl pentoxyethylene acetylene | 1.02499 | 0.213137 |
| 18 | tetradecyl isobutyrate | 1.02489 | 0.222997 |
| 19 | dimethyl trisulfide | 1.02428 | 0.218756 |
| 20 | naphthalene | 1.02421 | 0.217786 |
| 21 | (+)-isomenthone | 1.02411 | 0.313298 |
| 22 | cymene | 1.02403 | 0.230758 |
| 23 | di-tert-dodecyl disulfide | 1.02286 | 0.231832 |
| 24 | 2-heptanone | 1.02249 | 0.239725 |
| 25 | styrene | 1.02242 | 0.320197 |
| 26 | 3-octanone | 1.02182 | 0.231645 |
| 27 | calarene | 1.02059 | 0.255707 |
| 28 | p-menth-1(7),3-diene | 1.01903 | 0.323014 |
| 29 | α-terpineol | 1.01864 | 0.324472 |
| 30 | piperitone | 1.0185 | 0.320678 |
| 31 | 4-chlorobenzaldehyde | 1.01723 | 0.323218 |
| 32 | trans-2,4-decadienal | 1.01702 | 0.321436 |
| 33 | heptanal | 1.01697 | 0.320326 |
| 34 | furfuryl alcohol | 1.01632 | 0.253368 |
| 35 | 2,5-octanedione | 1.01508 | 0.318934 |
| 36 | trans-2-decenal | 1.01503 | 0.31921 |
| 37 | trans-2-dodecenal | 1.01502 | 0.319326 |
| 38 | isomenthone | 1.01476 | 0.321934 |
| 39 | menthone | 1.01463 | 0.322459 |
| 40 | p-cymene | 1.01449 | 0.324508 |
| 41 | trans-2-hexenal | 1.01429 | 0.324932 |
| 42 | menthofuran | 1.01427 | 0.32753 |
| 43 | 3-ethyl-2-methyl-1,3-hexadiene | 1.01409 | 0.326565 |
| 44 | menthol | 1.01348 | 0.339984 |
| 45 | α-curcumene | 1.01301 | 0.337613 |
| 46 | anethole | 1.01232 | 0.337635 |
| 47 | (E)-2-heptenal | 1.01218 | 0.314778 |
| 48 | (-)-pyrethrin D | 1.01108 | 0.358092 |
| 49 | nonanal | 1.01105 | 0.313119 |
| 50 | octaethylene glycol monododecyl ether | 1.01014 | 0.273288 |
| 51 | 4-carene | 1.00925 | 0.375167 |
| 52 | hexanal | 1.00925 | 0.309528 |
| 53 | (E,E)-2,4-nonadienal | 1.00894 | 0.307356 |
| 54 | octyl 2,2,3,3,3-pentafluoropropionate | 1.00816 | 0.378934 |
| 55 | 1,8-cineole | 1.00329 | 0.356162 |
| 56 | 1,3,5-trisilahexane | 1.00161 | 0.433412 |
| 57 | 1,3-di-tert-butylbenzene | 1.00131 | 0.317115 |


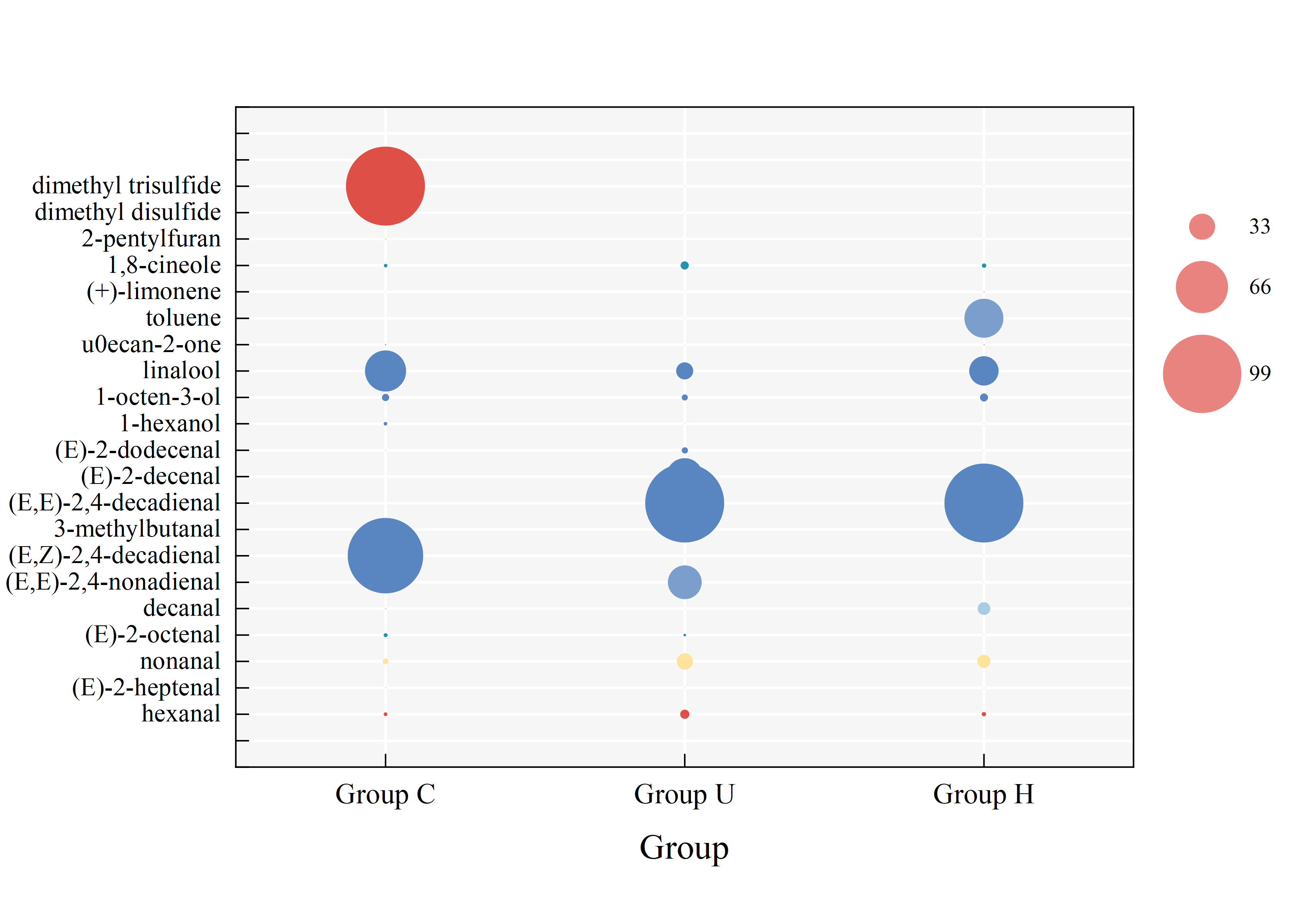


Figure S1 Bubble plot of OAV for three groups of chicken soup.
